# Multi-criteria assessment of optimization methods for controlling renewable energy sources in distribution systems

**DOI:** 10.1038/s41598-025-20339-5

**Published:** 2025-10-17

**Authors:** Ahmad Eid, Abdulrahman Alsafrani

**Affiliations:** Department of Electrical Engineering, College of Engineering, Qassim University, Buraidah, 52571 Saudi Arabia

**Keywords:** Optimization method, Algorithms, Distribution systems, Renewable energy source, Statistical assessment, Power losses, Voltage stability, Voltage deviation, Engineering, Electrical and electronic engineering

## Abstract

Numerous optimization techniques have recently been employed in the literature to enhance various electric power systems. Optimization algorithms help system operators determine the optimal location and capacity of any renewable energy source (RES) connected to a system, enabling them to achieve a specific goal and improve its performance. This study presents a novel statistical evaluation of 20 famous metaheuristic optimization techniques based on 10 performance measures. The performance measures comprise five power loss indices, three voltage profile indices, load flow calling frequency, and execution time. The evaluation involves 10 distribution systems of varying sizes to ensure an equitable comparison of the algorithm. The Friedman Ranking method evaluates algorithms based on performance metrics, yielding a specific score. Upon modeling all distribution systems, a composite ranking methodology is employed to categorize the algorithms into only four categories: excellent, very good, good, and fair. The study finalizes the ranking of all algorithms according to their overall assessment. The AEO, GWO, JS, PSO, MVO, BO, and GNDO algorithms attain ranks below 25%, thereby placing them in the highest category. The ALO, DA, FPA, SSA, YAYA, and SPO algorithms fall into the second category, with rankings ranging from 25 to 50%. The SMA and CGO algorithms are classified in the third group, with rankings between 50 and 75%. The analysis ultimately reveals that the algorithms CStA, HHO, AOA, GOA, and AOS are positioned in the lowest group, each achieving rankings beyond 75%. As comparison case studies, the proposed algorithms achieved a power loss of 87.164 kW for the 33-bus system, which is less than or equal to the published work. The same result is achieved with the 69-bus system, which has a power loss of 71.644 kW for most of the studied algorithms. Using the appropriate algorithms with distribution systems saves time and effort for the system operator, enhances performance, and increases the usability of optimization algorithms.

## Introduction

The global decline of fossil fuels and its detrimental effects on the environment and human health prompted the researcher to explore other energy sources, such as renewables, including solar and wind energy. Renewable energy sources (RESs) can be integrated into current distribution networks to alleviate the burden on transformers during peak demand periods. However, incorporating RESs may lead to increased losses in the system, a compromised voltage profile, decreased stability, or other shortcomings if not correctly chosen and connected. In this instance, the function of optimization techniques is realized^1–4^. The optimization algorithms can choose the correct place and size of any RES connected to a distribution system to achieve a specific objective, such as reducing system loss.

By providing a comprehensive understanding of their advantages and disadvantages, classifying optimization algorithms significantly improves the process of selecting the right one. It helps create solutions tailored to the specific needs of distribution systems. This systematic classification reduces the likelihood of incorrect rankings resulting from simplistic evaluations, allowing decision-makers to rely on a range of performance metrics. It also supports thorough assessments that consider practical challenges, ensuring the selected algorithms can effectively address real-world energy management problems. Furthermore, the classification framework facilitates the incorporation of new algorithms, thereby encouraging continuous improvements in selection methods. Ultimately, this structured approach promotes the adoption of more effective and efficient optimization strategies, resulting in improved energy management outcomes.

### Literature review

Many optimization algorithms have been adopted in the literature to optimize different distribution systems. In a recent work^5^, the authors adopted a logarithmic version of PSO to reduce losses in the IEEE 34-bus unbalanced system microgrid. The authors compared the results of the logarithmic PSO to those of different algorithms in many case studies. In^6^, the spotted hyena optimizer (SHO) was used to minimize the effects of integrating electric vehicle (EV) charging stations into distribution systems with the help of distributed generations (DGs) and static synchronous compensators (SSCs). The study examined the loss, stability, and reliability of the 69-bus system within a multi-objective (MO) optimization framework. Additionally, the study included various case studies and comparisons between different algorithms. In a similar study^7^, the authors applied the MOPSO algorithm to place the PV generator into the 9- and 15-bus systems using time-domain simulation in MATLAB/Simulink to maximize the power factor and voltage profiles. In^8^, a hybrid algorithm combining Firefly and Spider Monkey optimization was employed to minimize the operational expenses of a 33-bus microgrid, which includes electric vehicles, wind turbines, and photovoltaic power units. Another study^9^ adopted the DAOA and PSO algorithms to optimally allocate DG units to flatten the demand of unbalanced distribution systems while enhancing power quality. The study included three unbalanced systems, each with 10, 13, and 37 buses. The DAOA and PSO algorithms were compared in different case studies, including performance indicators of unbalanced voltage, power, and current factors.

In a recent study^10^, the Crow Search Algorithm (CSA) enhanced the radial 33-bus system by strategically positioning capacitor units to improve network performance through loss reduction and stability enhancement. The authors evaluated the system’s performance against six additional algorithms: PSO, ABC, GA, TLBO, and IWO. The authors in^11^ applied modified JAYA and GA algorithms to optimize the 33-, 47-, and 69-bus systems by integrating DG, capacitor banks, V2G, and EV charging stations. The study included the costs of optimized units and system performance enhancement during multiple case studies. In a recent study^12^, The Bonobo optimizer was employed in both single and multi-objective optimization frameworks to alleviate the demand fluctuations of the 69-bus system utilizing existing batteries and photovoltaic sources. The system was optimized throughout the day to demonstrate battery charging and discharging scenarios. Furthermore, the demands of residential, commercial, and industrial sectors were considered. The Pareto optimal front was selected using MOBO case studies, and the fuzzy logic function, together with TOPSIS approaches, were employed to identify the most favorable compromise options.

The authors of^13^ synthesized immune algorithms, moth flame optimization, and evolutionary strategies to reduce power loss in the 69- and 118-bus systems. The study included various distributed generation (DG) forms, I, II, and III, with single and multiple units. The authors of^14^ adopted the SMA and other algorithms to optimally allocate EVCS into the 33- and 69-bus systems. The research sought to minimize system losses and improve voltage profile and stability. Various DG, DSTATCOM, and battery energy storage system (BESS) units were utilized to meet the demand for electric vehicle charging stations (EVCS). The SMA was evaluated against other CSA, BA, and AVOA algorithms across several case studies. In another study^15^, the Honey Badger Algorithm (HBA) governed the wind turbine-battery energy storage system (BESS) units integrated with the 69-bus system to optimally manage three electric vehicle charging stations (EVCS) located at distinct sites within the system. The HBA minimized daily energy loss despite the unpredictable demand of EVCS and the state of charge of batteries, while enhancing the voltage profile and stability of the system.

Meanwhile, the study^16^ focused on the optimal integration of PV and DSTATCOM into radial 33- and 69-bus systems using the MVO algorithm to reduce the annual costs of PV and DSTATCOM devices as well as the operational costs of the networks. A comparative analysis of system performance was conducted utilizing various algorithms, including MVO, CGO, PSO, CSA, and VSA. The authors in^17^ integrated PV solar resources into the 33-bus system using the MOSMA algorithm to achieve different objectives. The study aimed to optimize the voltage profile and penetration level while minimizing power losses. The study considered various photovoltaic units ranging from one to five, with penetration levels reaching up to 300%. The MOSMA results were compared to those obtained using the NSGA-II, JAYA, and MOPSO algorithms. Another study^18^ adopted the Gorilla Troop Optimizer (GTO) to control eight EVCS allocated within the 108-bus system equipped with five groups of PV-BESS and WT-BESS. With the stochastic demand of the system and the state of charge of the batteries during the day, the GTO regulated each battery’s power to maintain the voltage and other constraints within acceptable limits.

In a recent study^19^, the MOHHO optimized the 69- and 118-bus systems to enhance the voltage profile and reduce system losses. The authors adopted the simplest aggregation method for the MO problem-solving. A similar study^20^ adopted the MODA to minimize the power loss, voltage variations, and total investment of reactive power of the 30- and 69-bus systems integrated with DG and reactive power sources. In a different work^21^, the authors developed and implemented the Enhanced Equilibrium Optimizer (EEO) to efficiently distribute PV-BESS units within the 69-bus system to minimize daily and annual energy losses. The study encompassed the simulation of the system over four distinct days to illustrate the summer, winter, spring, and autumn seasons. The Northern Goshawk Optimization (NGO)^22^ regulated the reactive power compensation produced by switching capacitors in the 33-bus system integrated with photovoltaic sources. Power losses were minimized and regulated, and annual net savings were optimized across the four seasons of the year. The Zebra Optimization Algorithm (ZOA)^23^ regulated different DG types and capacities to reduce power losses and enhance the voltage stability of the 33-bus system. The study included different scenarios with single and multiple DG units and types. Another study^24^ adopted the MOMVO for optimal allocation of PV and wind turbines and BESS units into the 33- and 141-bus systems using parallel processing and the Pareto front technique. The study simulated the systems for three days in the summer, winter, and spring seasons. Statistical analyses were performed on the different case studies. Other studies dealt with optimization algorithms, and their use can be explored in many literature sources such as^25–29^. The adopted optimization algorithms are listed in Table 1, along with their parameters and constants, sources of inspiration, and the year and publication source.

The categorization of current optimization algorithms utilized in distribution systems is crucial for the progression of energy management and distribution. By classifying these algorithms according to multiple criteria instead of just rating them, researchers and practitioners can gain a thorough understanding of their advantages and drawbacks^30–33^. This method facilitates a detailed assessment that takes into account various measures and factors, including system losses, voltage profile, speed of execution, load flow calling frequency, voltage stability, and relevance to real-world distribution system challenges. Establishing a systematic classification framework enables the proper evaluation of various optimization algorithms, ensuring that the selection of an algorithm is tailored to the specific needs of a distribution system. This systematic method not only averts deceptive rankings but also promotes informed decision-making, resulting in more effective and efficient optimization techniques in distribution system management.

**Table 1:** Adopted algorithms with their properties.

No.	Method	Parameters and constants	Inspiration	Year	Source	Ref.
1	PSO	$\:w=1,\:{c}_{1}={c}_{2}=$1.5	Swarm theory	2011	Conf.	^34^
2	FPA	$\:p=0.8,\:L,\:JK=rand$	Pollination process of flowers	2012	Journal	^35^
3	GWO	$\:{r}_{1},{r}_{2},\:{c}_{1},\:{c}_{2},\:{c}_{2}=rand$ $\:{A}_{1},{A}_{2},{A}_{3}=rand$	Hunting mechanism of wolves in nature	2014	Journal	^36^
4	ALO	$\:{r,r}_{1},\:{r}_{2},\:{r}_{3}=rand$	Nature-inspired behavior of ant lions	2015	Journal	^37^
5	DA	$\:a,c,f,e,s=rand$	Nature-inspired	2016	Journal	^38^
6	MVO	$\:{W}_{m}=0.2,\:{W}_{x}=1$ $\:{r}_{1},\:{r}_{2},\:{r}_{3}=rand$	Nature-inspired concepts in cosmology	2016	Journal	^39^
7	JAYA	$\:{{\varnothing}}_{1},\:{{\varnothing}}_{2}=rand$	Mathematical based	2016	Journal	^40^
8	GOA	$\:cMax=1,\:cMin=4\times\:{10}^{-5}$	Nature-inspired by grasshopper swarms	2017	Journal	^41^
9	SSA	$\:{c}_{1},\:{c}_{2},\:{c}_{2}=rand$	Nature-inspired behavior of salps	2017	Journal	^42^
10	AEO	$\:{r,r}_{1},\:{r}_{2},\:{r}_{3}=rand$, $\:a,\:u,\:v,\:c=rand$	Nature-inspired	2019	Journal	^43^
11	HHO	$\:r,q,e=rand$	Nature-inspired behavior of Harris Hawks	2019	Journal	^44^
12	GNDO	$\:a,b,c=rand$	normal distribution model	2020	Journal	^45^
13	SMA	$\:z=0.03$ $\:r,A,\:B=rand$	Oscillation mode of slime mould in nature	2020	Journal	^46^
14	CGO	$\:\alpha\:,\beta\:,\gamma\:=random$	Chaos theory	2021	Journal	^47^
15	JS	$\:{A}_{r},\:j=rand$	Nature-inspired behavior of jellyfish	2021	Journal	^48^
16	AOA	$\:\alpha\:=5,\:\mu\:=0.5$	Arithmetic operations	2021	Journal	^49^
17	AOS	$\:L=4,\:f=0.1$	Quantum mechanics	2021	Journal	^50^
18	CStA	$\:{r,r}_{1},\:{r}_{2},\:{r}_{3}=rand$	Formation of crystal structures	2021	Journal	^51^
19	SPO	$\:{I}_{d1},{I}_{d2},{I}_{d3},{I}_{d4}=rand$	Inspired by the art of painting colors	2022	Journal	^52^
20	BO	$\:pm=0.03,\:cab=1.25,\:csb=1.3,\:tsg=0.05,\:,\:rcp=0.0035$	Social behavior of Bonobos	2022	Journal	^53^

### Research gap

The No Free-Lunch Theorem asserts that no universal algorithm can be optimal for all problems. As a result, researchers have utilized several optimization methodologies. Researchers often use an optimization strategy to decrease or maximize a specific objective, typically one, two, or at most three, regardless of other critical system or problem metrics. Increasing the integration of renewable sources in distribution systems may lead to increased system losses or reduced stability. An analysis of the literature in the previous section and other available sources reveals a deficiency in explicit guidance for applying optimization methods in distribution systems. Each author chooses one or more optimization techniques to tackle a specific problem or achieve a goal. Ultimately, the readers or decision-makers are unable to identify the most advantageous choice among them. This paper introduces a ranking approach for 20 optimization algorithms, based on 10 performance measures, to address this research gap. Algorithms are assessed for applicability and scalability across 10 distribution systems of varying sizes, including small, medium, and large feeder buses. The evaluation framework is based on the statistical Friedman Ranking approach.

### Contribution

This study involved simulating, testing, and evaluating 20 algorithms. These algorithms encompass both traditional and contemporary methods utilized throughout various study domains, including power systems. The PSO algorithm is regarded as one of the oldest and most established algorithms, having emerged in 1995. The FPA and GWO were established in 2012 and 2014. The list comprises three algorithms introduced in 2015 (ALO, DA, MVO), two in 2017 (GOA, SSA), two in 2019 (AEO, HHO), five from 2020 (CGO, GNDO, JJS, SMA, SPO), and five from 2021 (AOA, AOS, BO, CStA, JAYA). These algorithms are selected due to their extensive application among power system researchers. The complete list of algorithms is simulated and evaluated according to 10 performance metrics utilizing the statistical method of Friedman Ranking. The 20 algorithms optimize 10 distribution systems, which range in size from 15 to 136 feeder buses, to assess several prominent systems and accurately rank the algorithms. The proposed technique evaluates the optimization algorithms for each distribution system, and a comprehensive assessment is also presented. The main contribution points of this work can be summarized as follows.

- *Comprehensive Evaluation*: This study uniquely simulates and analyzes twenty optimization algorithms, providing a robust comparison that encompasses both historical and cutting-edge techniques in the context of distribution systems.
- *Diverse Performance Metrics*: By employing ten performance metrics for the assessment, this research goes beyond the typical single or dual-objective evaluations found in existing literature, offering a multi-faceted view of algorithm performance.
- *Scalable System Modeling*: The evaluation is based on a diverse set of ten distribution systems, representing small, medium, and large-scale configurations. This approach enables a more comprehensive understanding of algorithm effectiveness across various operational contexts.
- *Statistical Rigor*: The application of the Friedman Ranking method provides a thorough and systematic assessment of the algorithms, consolidating results from various systems to deliver clear insights into their relative performance and robustness.

## Performance metrics

Selecting an appropriate optimization technique is crucial for complex situations, such as optimizing electrical distribution systems. Numerous authors and researchers have employed algorithms to optimize or minimize objective functions, often dealing with two or more objectives simultaneously in multi-objective optimization. The effectiveness of an optimization algorithm should not be solely dictated by its ability to accomplish a singular objective. The selected algorithm must possess sufficient flexibility to achieve various objectives. The efficacy of an optimization method must be evaluated using many measures. Key characteristics include convergence rate, solution accuracy (as measured by the standard deviation of different solutions), voltage profile of the system, scalability, and the time required to optimize the system. By evaluating the algorithm’s performance across several criteria, researchers can select the most suitable optimization approach for a problem, such as enhancing an electrical distribution system, leading to more efficient and effective solutions. This study evaluates the optimization technique using 10 performance metrics. The Friedman Ranking conducts the evaluation. The subsequent section examines the performance metrics adopted. The algorithm optimizes the system to minimize power loss, while also considering other vital metrics in the ranking process.

### Metrics of power losses

The power losses of a distribution system are calculated by summing the losses of the feeders due to their resistance and reactance^18,21,54^.

1 $$\:{P}_{L}=\sum\:_{i}^{{N}_{b}}{I}_{i}^{2}\times\:{R}_{i}$$

2 $$\:{Q}_{L}=\sum\:_{i}^{{N}_{b}}{I}_{i}^{2}\times\:{X}_{i}$$

Where $\:{P}_{L}$, $\:{Q}_{L}$ are the real and reactive losses of the system; $\:{R}_{i}$, $\:{X}_{i}$ are the branch resistance and reactance; $\:{I}_{i}$ is the ith branch current; $\:{N}_{b}$ is the number of branches.

After solving the system 50 times, the metrics of minimum, average, and maximum active loss ($\:{P}_{L,min}$, $\:{P}_{L,ave}$, $\:{P}_{L,max}$) are obtained with its standard deviation ($\:std$). The minimization of the power loss is the objective function ($\:{f}_{obj}$).

3 $$\:{f}_{obj}={P}_{L}$$

### Metrics of reactive power losses

The metric of reactive loss is the average of the reactive loss of the 50 system simulations.

4 $$\:{Q}_{La}=\sum\:_{k=1}^{{N}_{rx}}{Q}_{L,k}/{N}_{rx}$$

Where $\:{Q}_{L,k}$ is the kth run reactive loss.

### Metric of voltage deviation

The total voltage deviation (TVD) expresses how far the bus voltage is from the unity. As the algorithm runs 50 times, the average TVD is considered a performance metric^15,55,56^.

5 $$\:{TVD}_{a}=\sum\:_{k=1}^{{N}_{rx}}\sum\:_{i}^{{N}_{s}}\left|1-\left|{V}_{i}\right|\right|/{N}_{rx}$$

Where $\:{N}_{s}$ is the number of buses; $\:{V}_{i}$ is the ith bus voltage; $\:{N}_{rx}$ is the maximum number of runs (50).

### Metric of voltage stability

The voltage stability index (SI) is calculated as follows^12,57–59^.

6 $$\:SI=1-{\left[2\left({P}_{i}{R}_{i}+{Q}_{i}{X}_{i}\right)-{V}_{k}^{2}\right]}^{2}-4{S}_{i}^{2}{Z}_{i}^{2}$$

7 $$\:{SI}_{a}=\sum\:_{n=1}^{{N}_{rx}}{SI}_{n}/{N}_{rx}$$

$$\:{Z}_{i}={R}_{i}+{jX}_{i}$$, $$\:{S}_{i}={P}_{i}+{jQ}_{i}$$(8)

Where $\:{SI}_{a}$ is the average of SI over the 50 runs; $\:{Z}_{i}$ is the complex impedance of the branch; $\:{S}_{i}$ is the complex power at the receiving end of the branch; $\:{V}_{k}$ is the voltage at the sending end of the branch.

### Metric of minimum voltage

The minimum voltage metric is the average minimum voltage recorded during the 50 solutions of the system.

9 $$\:{V}_{La}=\sum\:_{k=1}^{{N}_{rx}}{V}_{L,k}/{N}_{rx}$$

10 $$\:{V}_{L}=\text{min}\left({V}_{i}\right),\:i=\text{1,2},\dots\:,\:{N}_{s}$$

Where $\:{V}_{L,k}$ is the minimum voltage of the kth run; $\:{V}_{i}$ is the voltage at the ith bus.

### Metric of execution time

**Fig. 1:**
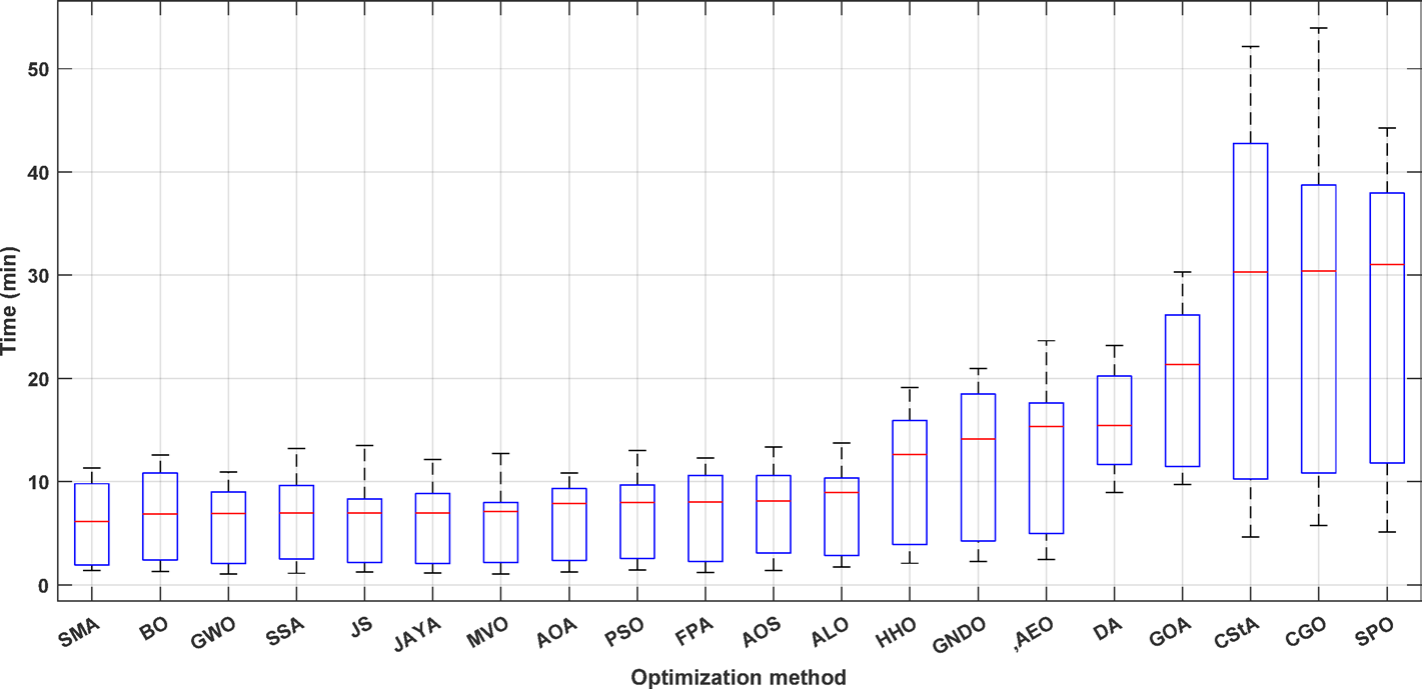
Variation of the execution time of algorithms solving all distribution systems for 50 different runs.

Execution time varies among algorithms. The shorter the execution time, the better. A faster algorithm allows for online operation monitoring. Additionally, fast algorithms can handle extensive data more efficiently with fewer resources compared to slower ones. In this study, the execution time is recorded over 50 runs for each algorithm on every system and used as a performance metric. Figure 1 displays a boxplot of execution times for 20 algorithms optimizing 10 distribution systems, arranged by median value. The first twelve algorithms (SMA, BO, GWO, SSA, JS, JAYA, MVO, AOA, PSO, FPA, AOS, and ALO) require, on average, under 8 min for 50 runs (less than 10 min for the medians). In contrast, the following five algorithms (HHO, GNDO, AEO, DA, and GOA) complete 50 runs across the 10 systems in an average of 20 min or less. Only the final three algorithms (CStA, CGO, SPO) require an average of 28 min or less for 50 runs per system across the 10 systems. In terms of average execution times, SMA is the fastest, while CGO is the slowest.

### Metric of load flow calling frequency

**Fig. 2:**
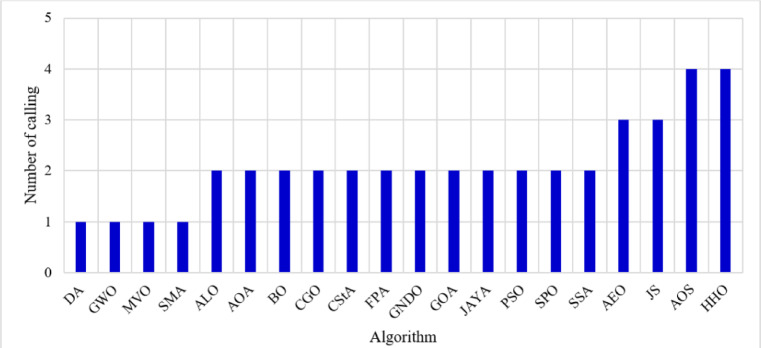
Calling frequency of the objective function for each algorithm.

The load flow algorithm resolves the distribution system and, consequently, the objective functions. Algorithms differ based on their architecture and the frequency with which they invoke the objective function. Typically, an algorithm that executes fewer objective function calls operates more swiftly than one that invokes the objective function multiple times. The frequency of objective function evaluations for the 20 algorithms is presented in Fig. 2. Most known methods evaluate the objective functions twice. Four algorithms, namely DA, GWO, MVO, and SMA, invoke the objective function alone once. Conversely, AEO and JS algorithms require three evaluations to achieve the objective, but AOS and HHO necessitate four assessments of the objective function.

## Proposed methodology of assessment

**Fig. 3:**
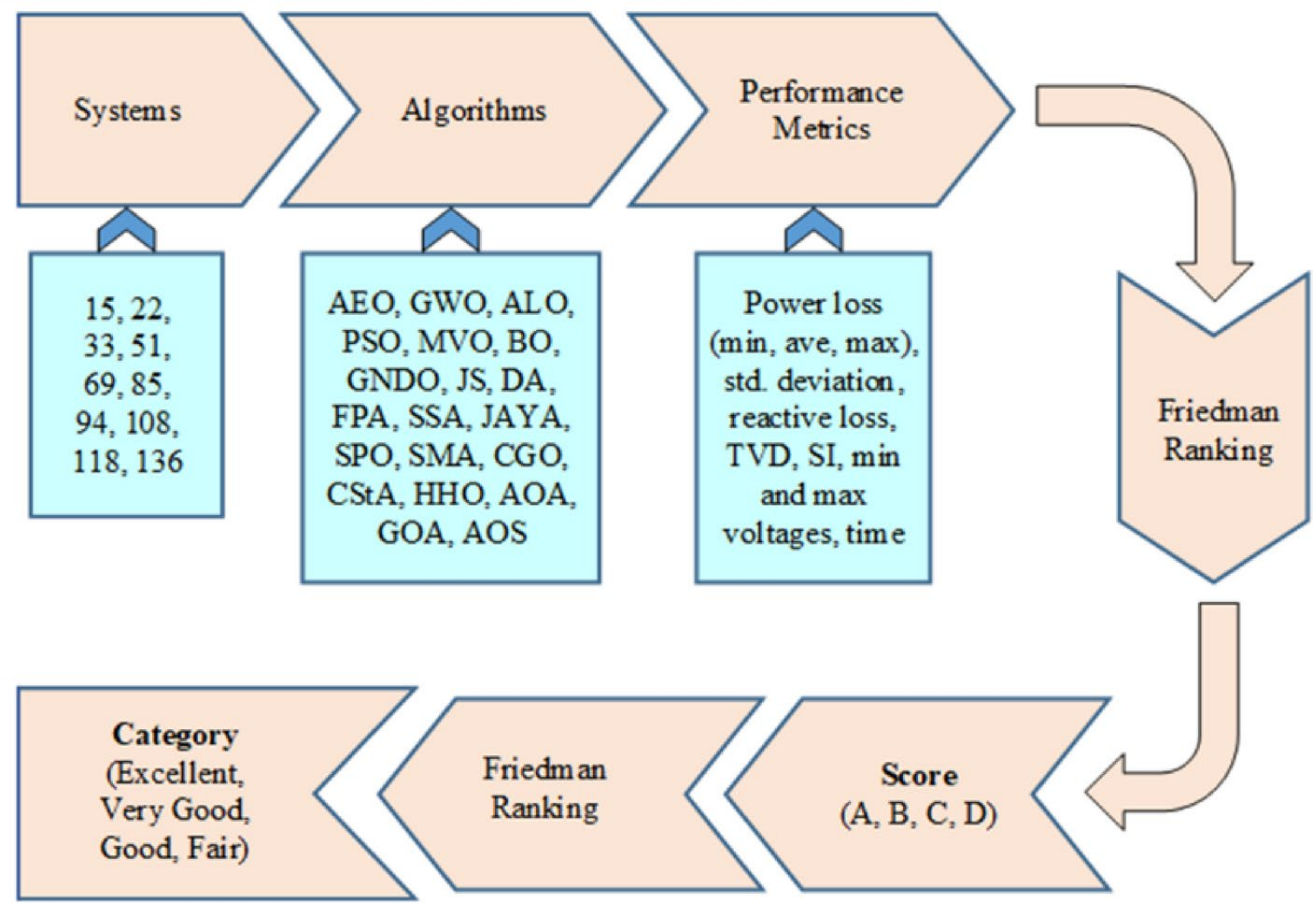
Layout of the proposed assessment methodology.

Numerous metaheuristic algorithms exist, and researchers continually innovate new varieties and hybrids to address diverse and challenging optimization problems. Moreover, many of them are adopted in the area of distribution systems when integrated with renewable energy sources. The assessment method follows a series of steps, as illustrated in Fig. 3. The 20 optimization algorithms are selected to optimize the performance of 10 distribution systems. The algorithms encompass a range of well-established and emerging algorithms. On the other hand, different distribution systems are simulated and optimized to assess the algorithms. The systems are available in small, medium, and large sizes. These systems are 15-, 22-, 33-, 51-, 69-, 85-, 94-, 108-, 118-, and 136-bus. The considered algorithms are listed, in alphabetical order, AEO, ALO, AOA, AOS, BO, CGO, CStA, DA, FPA, GNDO, GOA, GWO, HHO, JAYA, JS, MVO, PSO, SMA, SPO, and SSA. Each algorithm executes the system 50 times to facilitate statistical analysis and comparison, yielding more reliable statistical outcomes. Each execution involves invoking load flow solutions, setting the maximum number of iterations, and modifying algorithm parameters. All simulations aim to reduce the power loss in the distribution system. As a result, 50 values for the objective function are gathered to ascertain the statistical parameters, including the minimum, maximum, average, and standard deviation of power loss. Furthermore, six additional performance measures are documented: reactive loss, total voltage deviation (TVD), stability index (SI), minimum and maximum bus voltages within the system, and the duration required to complete the 50 runs.

**Fig. 4:**
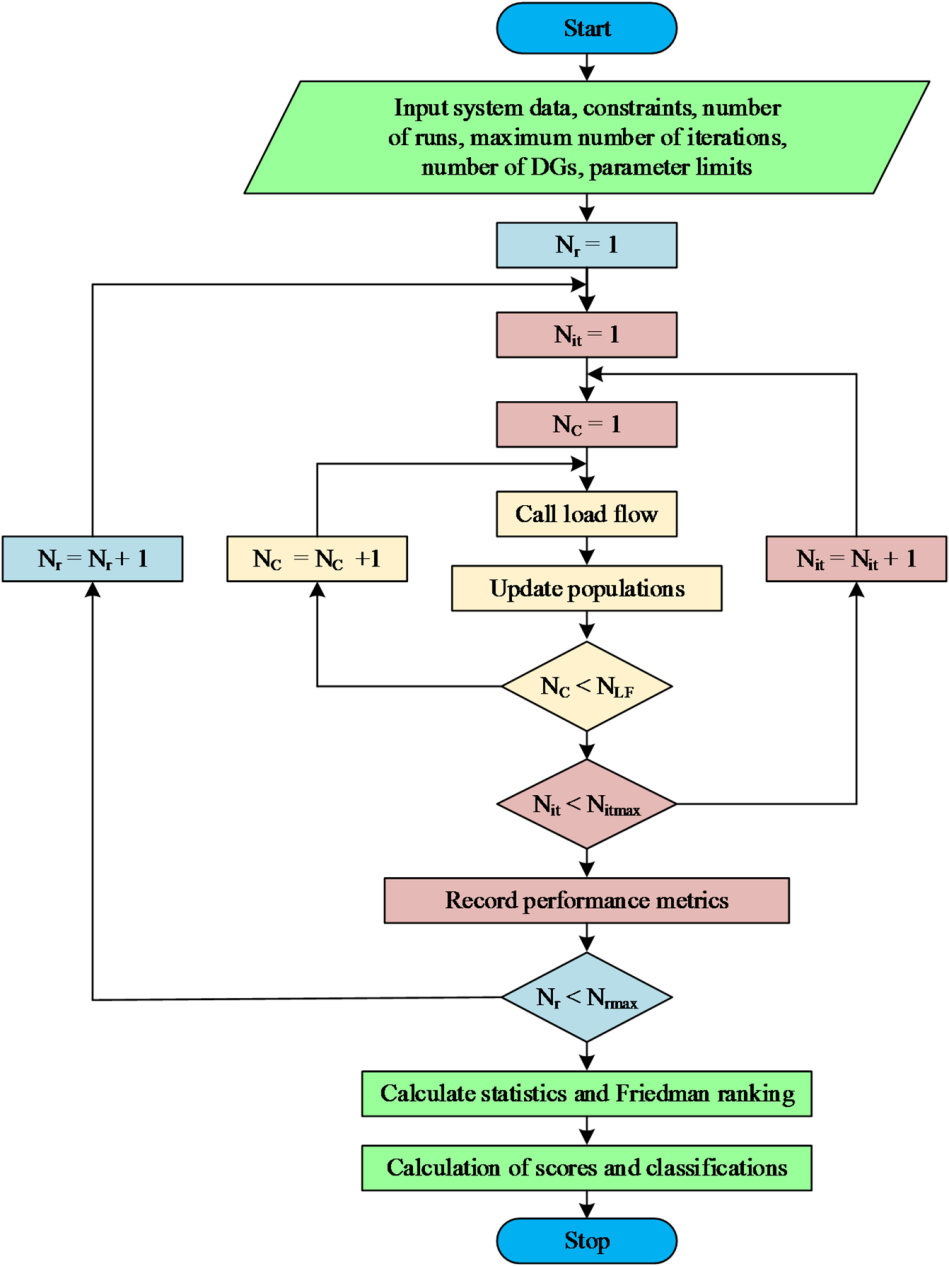
Flow chart of the proposed methodology.

Upon gathering data from all systems simulated by the various algorithms, the Friedman ranking method is employed to evaluate the 20 algorithms for each distribution system. The Friedman test is used to compare 20 algorithms that address the same system. The Friedman test output is normalized regarding the minimum value to determine the score of each algorithm. The assessment process establishes the hierarchy of each performance metric for every examined distribution system. The optimization algorithms are rated as A, B, C, or D, with A being the highest quality and D the lowest. The optimization algorithm is assigned an A score for rankings under 25%, a B score from 25% to 50%, a C score from 50% to 75%, and a D score from 75% but under 100%. The algorithmic score varies across different systems. Consequently, a method that achieves an A score for one system does not ensure the same score when applied to a different system. The ultimate ranking of each algorithm is contingent upon the scores obtained from the 10 distribution systems. The 20 algorithms are categorized under four classifications: Excellent, Very Good, Good, and Fair. The classifications are derived from the aggregate and kind of scores obtained by the algorithm from the 10 performance metrics of the 10 distribution systems. A flow chart displaying the complete solution steps, ranking, and classification processes is shown in Fig. 4. The suggested methodology includes three distinct loops. The innermost loop operates on the load flow solution of the distribution system.

The variables $\:{N}_{C}$ and $\:{N}_{LF}$ are the current and the maximum number of load flow calling routines. The intermediate loop examines the presumed quantity of iterations. The variables $\:{N}_{it}$ and $\:{N}_{itmax}$ are the current iteration and the maximum number of iterations. The outermost loop spans the number of execution times that the algorithm executes. The variable $\:{N}_{r}$ and $\:{N}_{rmax}$ are the current and maximum number of runs. Upon completing the 50 runs, the system data are compiled, and statistics are computed for each method to determine the lowest, maximum, average, and standard deviations of the power loss. Additional performance indicators are also gathered. The subsequent phase involves computing the Friedman ranking and the scores of the algorithms. In the final stage, the classifications of the 20 algorithms are shown according to the performance scores attained using the 10 distribution methods.

## Simulations and observations

This section evaluates 20 optimization methods in simulating 10 distribution systems of varying sizes: small, medium, and large. The evaluation procedure relies on 10 quantified parameters representing the mean performance values from 50 runs of each algorithm. Every algorithm runs for 100 iterations and is considered the maximum iteration number with a population of 100 agents. Furthermore, each algorithm’s 50 runs are conducted five times, with the optimal result selected and documented. This study encompasses 10 distribution systems: the 15, 22, 33, 51, 69, 85, 94, 108, 118, and 136 bus systems. The evaluation of the optimization techniques is predicated on the mean of the performance metrics, including the minimum, average, and maximum power loss, their standard deviation, reactive power loss, total voltage deviation (TVD), stability index (SI), minimum bus voltage, duration of 50 iterations, and the frequency of load flow calls. As time is used as a performance metric, all simulations are run on MATLAB on the same laptop with the following specifications: Windows 11, Core i7, 2 GHz processor, and 8 GB RAM, running a 64-bit operating system. The ranking of each performance metric is calculated using the rank function, where values are ranked from lowest to highest. The average is considered whenever two or more ranks have the same value. After ranking all metrics, the total ranking of the algorithm is calculated, and the score is returned. In the following sections, all systems are considered and explored.

### Performance of the 15-bus system

**Fig. 5:**
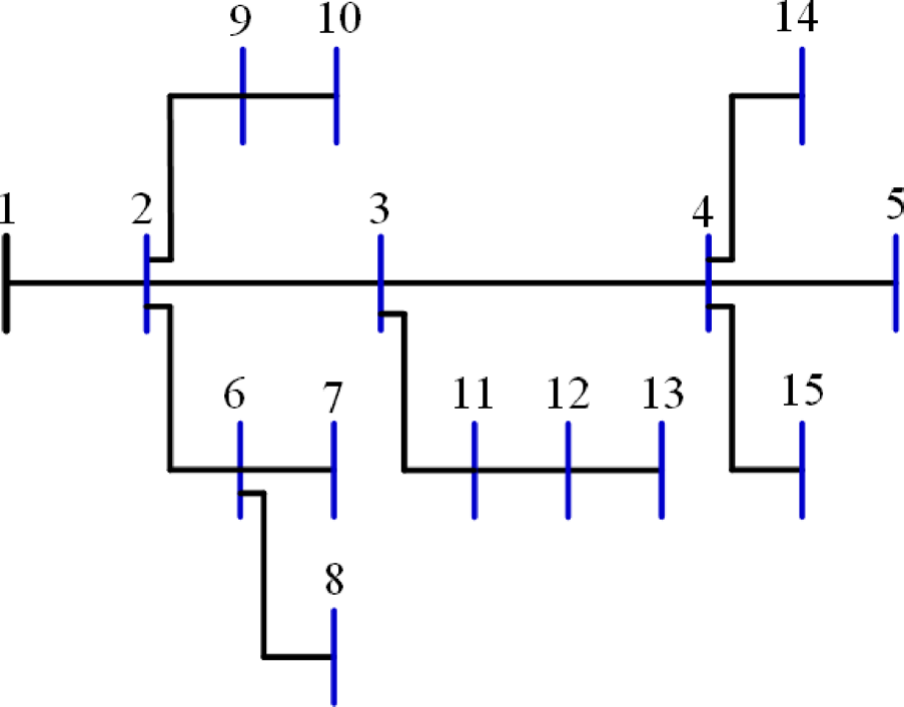
Layout of the 15-bus system.

The 15-bus distribution system is the smallest system studied in this research. The system has a total demand of $\:1226.4+\:j1251.1785$ kVA^60^ with a rated voltage of 11 kV, the system has a layout as shown in Fig. 5. The 20 algorithms optimize the system by integrating two DGs to reduce the losses to a minimum. The performance metrics are recorded and ranked. The performance metrics for the system, along with other information, are listed in Table 2. Besides the 10-performance metrics, Table 2 shows the upper voltage limit ($\:{V}_{U}$), the optimal DG sizes and sites. It is worth mentioning here that the optimal sites of DGs are the most common sites of the 50 runs of each algorithm. Also, the listed time is for the completion of the 50 runs. The $\:std$ refers to the standard deviation of the power loss of the 50 solutions. $\:{P}_{L,min}$, $\:{P}_{L,ave}$, $\:{P}_{L,max}$ refer to the minimum, average, and maximum power losses during the 50 runs while $\:{Q}_{L}$ is the reactive losses. The table arranges all algorithms according to their ranking and scores, with the best at the top.

**Fig. 6:**
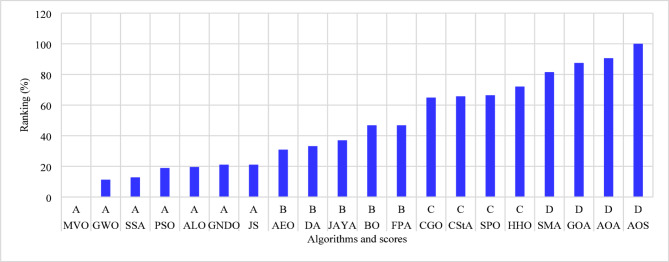
Ranking of algorithms and their scores with the 15-bus system.

The percentage ranking is calculated from 200 (10 systems times 20 algorithms), and the normalized ranking of the algorithms is shown in Fig. 6. Notably, the best algorithm has the lowest normalized ranking, which is zero. In contrast, the worst algorithm has a normalized ranking of 100%. Thus, the MVO algorithm achieved the best score among the 20 algorithms, while the AOS algorithm is the worst. The algorithms MVO, GWO, SSA, PSO, ALO, GNDO, and AEO have attained a score of (A) Algorithms AEO, DA, AYA, BO, and FPA are ranked in second place, achieving a score of (B) The algorithms CGO, CStA, SPO, and HHO attained a third-place ranking with a score of (C) The algorithms with the lowest rank, receiving a score of D, include SMA, GOA, AOA, and AOS.

**Table 2:** Performance parameters of the 15-bus system with different algorithms.

Method	$\:{\varvec{P}}_{\varvec{L},\varvec{m}\varvec{i}\varvec{n}}$ (kW)	$\:{\varvec{P}}_{\varvec{L},\varvec{a}\varvec{v}\varvec{e}}$ (kW)	$\:{\varvec{P}}_{\varvec{L},\varvec{m}\varvec{a}\varvec{x}}$ (kW)	$\:\varvec{S}\varvec{t}\varvec{d}$	$\:{\varvec{Q}}_{\varvec{L}}$ (kVar)	$\:\varvec{T}\varvec{V}\varvec{D}$ (pu)	$\:\varvec{V}\varvec{S}\varvec{I}$ (pu)	$\:{\varvec{V}}_{\varvec{L}}$ (pu)	$\:{\varvec{V}}_{\varvec{U}}$ (pu)	Time (min)
MVO	33.251	33.251	33.251	0.0E + 00	30.292	0.336	0.129	0.9651	1	1.07
GWO	33.251	33.251	33.251	0.0E + 00	30.292	0.336	0.129	0.9651	1	1.07
SSA	33.251	33.251	33.251	0.0E + 00	30.292	0.336	0.129	0.9651	1	1.12
PSO	33.251	33.251	33.251	0.0E + 00	30.292	0.336	0.129	0.9651	1	1.43
ALO	33.251	33.251	33.251	0.0E + 00	30.292	0.336	0.129	0.9651	1	1.72
GNDO	33.251	33.251	33.251	0.0E + 00	30.292	0.336	0.129	0.9651	1	2.28
JS	33.251	33.251	33.251	0.0E + 00	30.292	0.336	0.129	0.9651	1	1.24
AEO	33.251	33.251	33.251	5.07E-16	30.292	0.336	0.129	0.9651	1	2.46
DA	33.251	33.350	35.144	6.2E-03	30.379	0.335	0.129	0.9653	1	8.93
JAYA	33.251	33.251	33.251	0.0E + 00	32.075	0.346	0.130	0.9650	1	1.13
BO	33.251	33.266	34.033	1.8E-03	30.305	0.336	0.129	0.9651	1	1.27
FPA	33.251	33.280	33.431	5.5E-04	30.323	0.337	0.130	0.9651	1	1.19
CGO	33.251	33.251	33.251	0.0E + 00	41.233	0.466	0.158	0.9568	1	6.50
CStA	33.251	33.257	33.287	1.1E-04	31.590	0.356	0.132	0.9643	1	4.63
SPO	33.251	33.368	35.337	7.6E-03	33.287	0.334	0.131	0.9647	1	5.14
HHO	33.251	33.398	35.194	6.8E-03	30.409	0.339	0.130	0.9649	1	2.09
SMA	33.251	33.526	35.731	1.1E-02	50.246	0.563	0.184	0.9494	1	1.39
GOA	33.257	34.128	36.659	1.3E-02	30.986	0.347	0.130	0.9648	1	9.71
AOA	33.305	35.586	37.738	1.9E-02	32.372	0.382	0.138	0.9625	1	1.23
AOS	33.258	34.460	36.384	1.5E-02	35.953	0.409	0.146	0.9604	1	1.39

Table 2 shows the results of 20 optimization methods. Each method is evaluated based on parameters such as minimum, average, and maximum power loss, standard deviation, reactive power loss, convergence time, TVD, and the lower and upper voltage limits across 50 runs. The results indicate that while some methods produce similar outcomes, there are performance differences among various optimization techniques. This suggests that each method has its strengths and weaknesses in system optimization. The minimal standard deviation values imply that the methods tend to converge to nearly the same result in each run. MVO and GWO are the fastest algorithms, completing 50 runs in 1.07 min. Conversely, the slowest are DA and GOA, taking 8.93 and 9.71 min, respectively. The upper voltage limit remains constant at 1.0 pu for all methods. The lower voltage values are nearly identical, except for the SMA and CGO algorithms.

### Performance of the 22-bus system

**Fig. 7:**
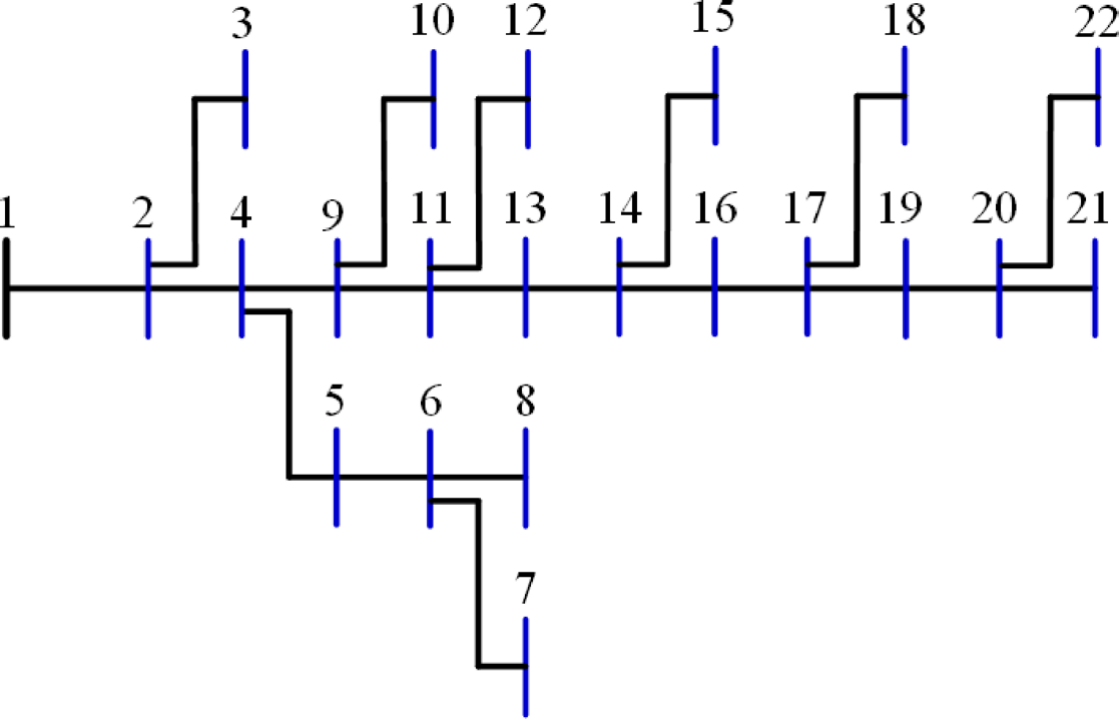
Layout of the 22-bus system.

**Fig. 8:**
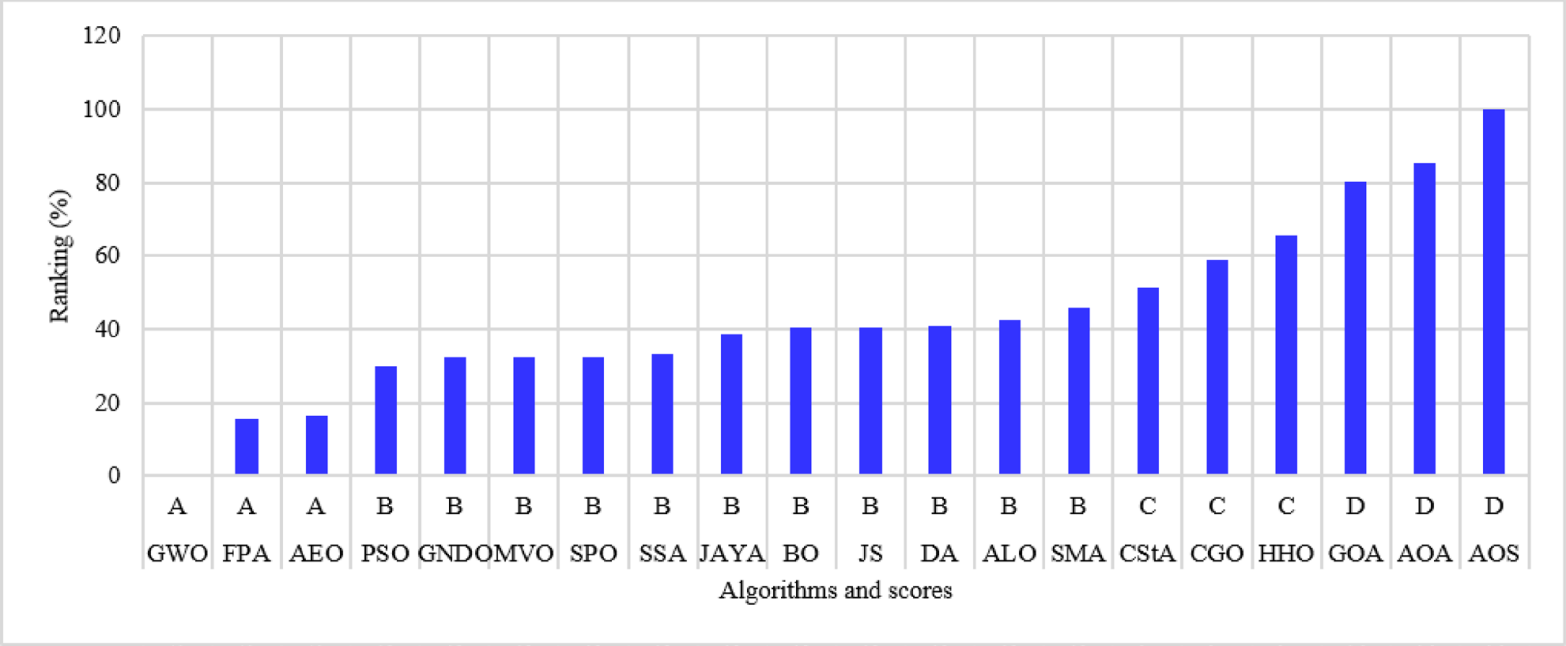
Ranking of algorithms and their scores with the 22-bus system.

The 22-bus system has a total demand of $\:662.311+j657.4$ kVA^61^ with a rated voltage of 11 kV, its schematic layout is shown in Fig. 7. The system is optimized with two DGs using the 20 algorithms to minimize the system loss. The performance metrics, along with other parameters, are listed in Table 3. The algorithms’ percentage rankings and scores are calculated and shown in Fig. 8. It is observed that the GWO method is superior, achieving an A score with a normalized rank of zero, indicating the lowest rank. In this system, three algorithms attained a rating below 25%: GWO, FPA, and AEO. The algorithms ranked second-best with a score of B include PSO, GNDO, MVO, SPO, SSA, JAYA, BO, JS, DA, ALO, and SMA. The algorithms CStA, CGO, and HHO attained a C score, whereas GOA, AOA, and AOS received the lowest score of D.

**Table 3:** Performance parameters of the 22-bus system with different algorithms.

Method	$\:{\varvec{P}}_{\varvec{L},\varvec{m}\varvec{i}\varvec{n}}$ (kW)	$\:{\varvec{P}}_{\varvec{L},\varvec{a}\varvec{v}\varvec{e}}$ (kW)	$\:{\varvec{P}}_{\varvec{L},\varvec{m}\varvec{a}\varvec{x}}$ (kW)	$\:\varvec{S}\varvec{t}\varvec{d}$	$\:{\varvec{Q}}_{\varvec{L}}$ (kVar)	$\:\varvec{T}\varvec{V}\varvec{D}$ (pu)	$\:\varvec{V}\varvec{S}\varvec{I}$ (pu)	$\:{\varvec{V}}_{\varvec{L}}$ (pu)	$\:{\varvec{V}}_{\varvec{U}}$ (pu)	Time (min)
GWO	8.468	8.468	8.468	5.09E-07	4.332	0.125	0.023	0.9904	1	1.59
FPA	8.468	8.472	8.484	4.49E-03	4.334	0.125	0.023	0.9904	1	1.65
AEO	8.468	8.468	8.468	7.00E-15	4.332	0.125	0.023	0.9904	1	2.79
PSO	8.468	8.473	8.514	1.51E-02	4.335	0.125	0.024	0.9905	1	1.8
GNDO	8.468	8.473	8.514	1.39E-02	4.335	0.125	0.024	0.9905	1	2.9
MVO	8.468	8.482	8.514	2.16E-02	4.340	0.126	0.024	0.9907	1	1.48
SPO	8.468	8.477	8.514	1.85E-02	4.800	0.103	0.021	0.9923	1.0003	6.50
SSA	8.468	8.480	8.514	2.01E-02	4.339	0.126	0.024	0.9906	1	1.41
JAYA	8.468	8.468	8.468	1.68E-05	4.451	0.133	0.025	0.9897	1	1.34
BO	8.468	8.484	8.514	2.15E-02	4.340	0.126	0.024	0.9907	1	1.55
JS	8.468	8.468	8.468	3.69E-05	4.427	0.130	0.025	0.9901	1	1.37
DA	8.468	8.484	8.544	2.04E-02	4.341	0.125	0.024	0.9907	1	9.31
ALO	8.468	8.479	8.520	2.06E-02	4.338	0.126	0.024	0.9906	1	2.09
SMA	8.468	8.468	8.468	1.62E-07	6.303	0.221	0.042	0.9831	1	1.84
CStA	8.468	8.472	8.482	4.01E-03	4.436	0.127	0.026	0.9910	1.0001	5.31
CGO	8.468	8.468	8.468	5.67E-15	6.354	0.247	0.048	0.9807	1.0001	5.74
HHO	8.468	8.495	8.563	2.53E-02	4.346	0.127	0.025	0.9908	1	2.50
GOA	8.469	8.532	8.644	5.06E-02	4.365	0.127	0.025	0.9907	1	11.47
AOA	8.496	8.644	8.945	9.59E-02	4.422	0.133	0.026	0.9902	1	1.49
AOS	8.468	8.551	8.654	4.94E-02	5.091	0.146	0.030	0.9892	1.0001	1.76

Examining Table 3 highlights several key points. According to the findings, there are variations in performance among the various optimization strategies, despite specific methods producing results that are comparable to one another. It can be deduced from this that every strategy has both advantages and disadvantages when it comes to optimizing the system. The very low standard deviation values suggest that the methods tend to converge to nearly the same result in each run. JAYA is the fastest algorithm, completing 50 runs in 1.34 min. In contrast, DA and GOA are the slowest, taking 9.31 and 11.47 min, respectively. The upper voltage limit remains steady at 1.0 pu for all methods except for four algorithms, which have slightly higher values. The lower voltage values are nearly identical.

### Performance of the 33-bus system

**Fig. 9:**
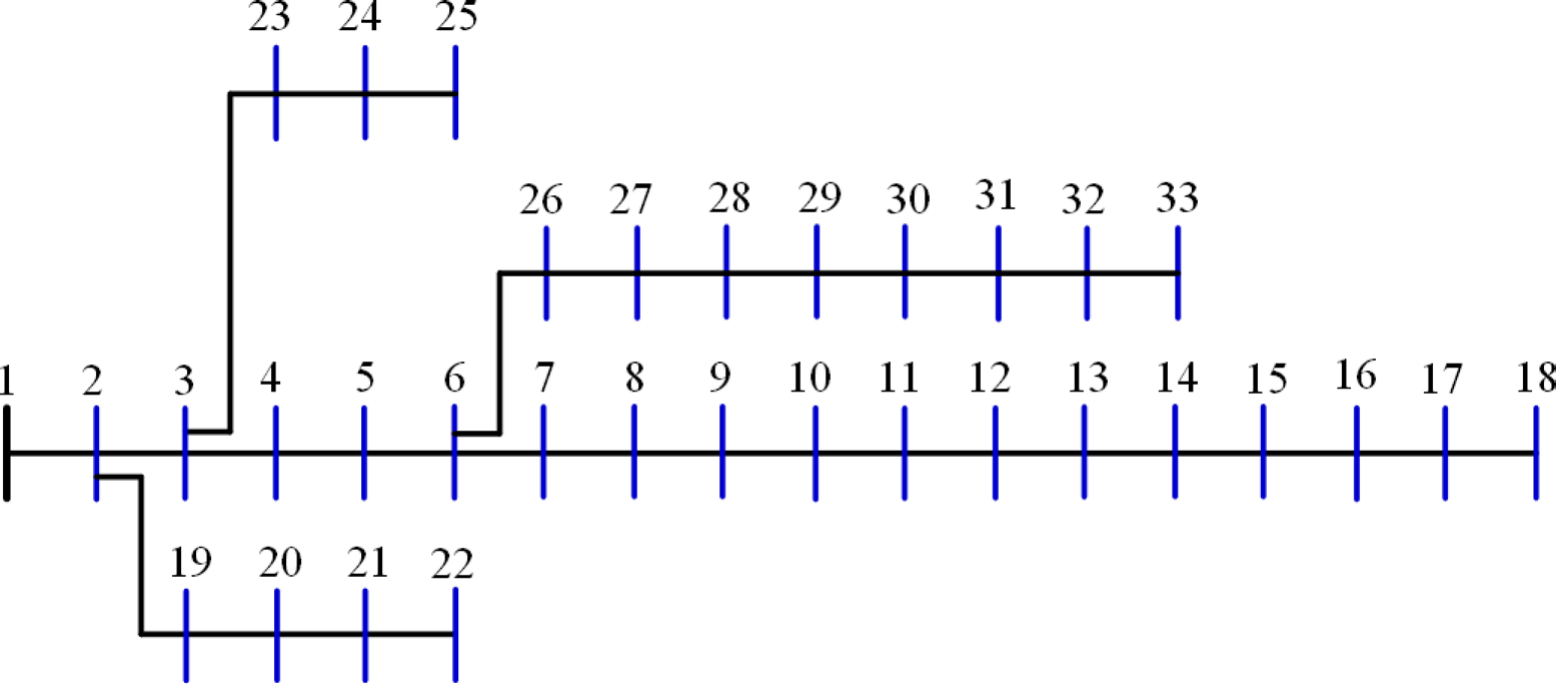
Layout of the 33-bus system.

**Fig. 10:**
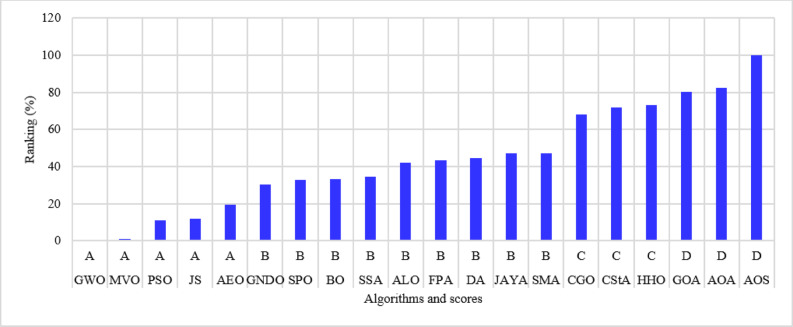
Ranking of algorithms and their scores with the 33-bus system.

The third studied system is the 33-bus system shown in Fig. 9. The system has a total demand of $\:3715+j2300$ kVA and a rated voltage of 12.66 kV^62^. The algorithms optimize the system using two DGs to minimize the system loss and run 50 times. The performance parameters are recorded and listed in Table 4. The percentage ranking and scores of the algorithms are shown in Fig. 10. The algorithms for the A score in this system include GWO, MVO, PSO, JS, and AEO. Nine algorithms scored second-place B: GNDO, SPO, BO, SSA, ALO, FPA, DA, JAYA, and SMA. Thirdly, three algorithms exhibit a C score: CGO, CStA, and HHO. Three algorithms, GOA, AOA, and AOS, occupy the lowest rank of score D.

**Table 4:** Performance parameters of the 33-bus system with different algorithms.

Method	$\:{\varvec{P}}_{\varvec{L},\varvec{m}\varvec{i}\varvec{n}}$ (kW)	$\:{\varvec{P}}_{\varvec{L},\varvec{a}\varvec{v}\varvec{e}}$ (kW)	$\:{\varvec{P}}_{\varvec{L},\varvec{m}\varvec{a}\varvec{x}}$ (kW)	$\:\varvec{S}\varvec{t}\varvec{d}$	$\:{\varvec{Q}}_{\varvec{L}}$ (kVar)	$\:\varvec{T}\varvec{V}\varvec{D}$ (pu)	$\:\varvec{V}\varvec{S}\varvec{I}$ (pu)	$\:{\varvec{V}}_{\varvec{L}}$ (pu)	$\:{\varvec{V}}_{\varvec{U}}$ (pu)	Time (min)
GWO	87.164	87.164	87.164	3.30E-05	59.773	0.677	0.120	0.9685	1	2.06
MVO	87.164	87.164	87.164	2.93E-06	59.773	0.677	0.120	0.96851	1	2.17
PSO	87.164	87.164	87.164	8.87E-14	59.773	0.677	0.120	0.96851	1	2.56
JS	87.164	87.164	87.164	8.11E-08	59.773	0.677	0.120	0.96851	1	2.16
AEO	87.164	87.164	87.164	7.06E-14	59.773	0.677	0.120	0.9685	1	4.98
GNDO	87.164	87.165	87.248	1.20E-02	59.773	0.677	0.120	0.9684	1	4.27
SPO	87.164	87.164	87.164	5.04E-14	73.072	0.655	0.126	0.96621	1.0027	11.80
BO	87.164	87.170	87.248	2.32E-02	59.773	0.677	0.121	0.9682	1	2.39
SSA	87.164	87.177	87.248	3.13E-02	59.772	0.677	0.122	0.96785	1	2.50
ALO	87.164	87.199	87.248	4.22E-02	59.771	0.678	0.125	0.9668	1	2.85
FPA	87.164	87.302	87.568	9.99E-02	59.908	0.675	0.125	0.9669	1	2.26
DA	87.164	87.248	88.419	1.97E-01	59.830	0.674	0.123	0.9673	1	11.66
JAYA	87.164	87.164	87.165	4.46E-04	74.613	0.861	0.180	0.95078	1.0005	2.05
SMA	87.164	87.164	87.164	4.04E-07	102.140	1.239	0.225	0.93618	1.0004	2.30
CGO	87.164	87.164	87.167	5.48E-04	97.531	1.123	0.242	0.9325	1.0036	10.85
CStA	87.164	87.283	87.447	7.88E-02	67.055	0.749	0.153	0.9589	1.0005	10.25
HHO	87.164	88.590	92.733	1.80E + 00	61.012	0.684	0.135	0.96407	1	3.90
GOA	87.166	89.585	97.859	2.48E + 00	61.764	0.687	0.141	0.9623	1	10.14
AOA	87.631	92.974	98.944	2.78E + 00	64.691	0.698	0.146	0.9610	1	2.37
AOS	87.344	90.573	97.984	2.53E + 00	101.830	0.931	0.196	0.9462	1.0094	3.08

Upon closer inspection, Table 4 reveals several essential features. It can be deduced from this that every strategy has both advantages and disadvantages when it comes to optimizing the system. The extremely low values of the standard deviation indicate that the approaches tend to converge to nearly the same outcome in every application, except for the HHO, GOA, AOA, and AOS algorithms. JAYA and GWO are the algorithms that finish 50 runs in 2.05 and 2.06 min, making them the quickest algorithms. DA and SPO, on the other hand, are the slowest algorithms, taking 11.66 and 11.80 min, respectively. Except for six algorithms, which have somewhat higher values, the upper voltage limit remains constant at 1.0 pu for all approaches. The values of the lower voltage are almost identical, except for CGO and SMA, which achieve lower values, as reflected in the TVD values.

### Performance of the 51-bus system

**Fig. 11:**
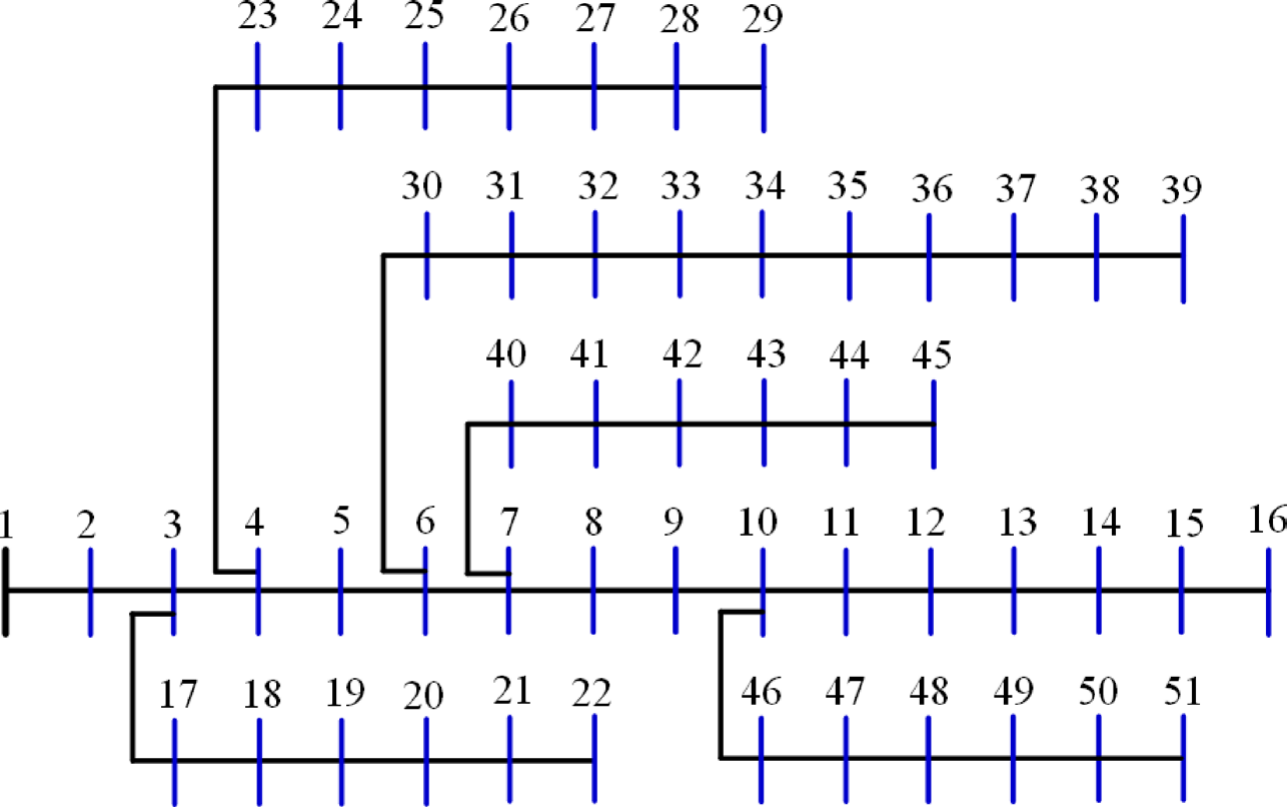
Layout of the 51-bus system.

**Fig. 12:**
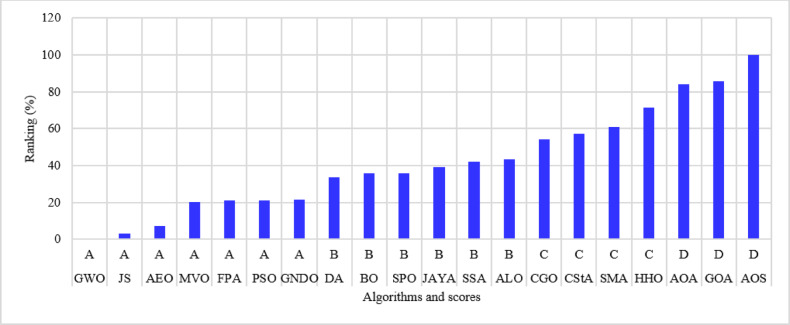
Ranking of algorithms and their scores with the 51-bus system.

The 51-bus system is rated at 11 kV with a total demand of $\:2463+j1569$ kVA^63^. The system’s layout is shown in Fig. 11. Optimizing the system involves assigning two DGs to distinct algorithms to reduce the amount of power lost across the system whenever possible. The statistical calculations are performed by each method 50 times, as many times as they were before. Table 5 lists the components that contribute to system performance. In the 51-bus system, seven algorithms have achieved a score under 25%. The ideal algorithms, listed in descending order, are GWO, JS, AEO, MVO, FPA, PSO, and GNDO, each attaining an A score, as shown in Fig. 12. The algorithms DA, BO, SPO, JAYA, SSA, and ALO received second place with a score of B. The C scoring algorithms are CGO, CStA, SMA, and HHO. Ultimately, the AOA, GOA, and AOS algorithms occupy the lowest rank, having attained the lowest score of D.

**Table 5:** Performance parameters of the 51-bus system with different algorithms.

Method	$\:{\varvec{P}}_{\varvec{L},\varvec{m}\varvec{i}\varvec{n}}$ (kW)	$\:{\varvec{P}}_{\varvec{L},\varvec{a}\varvec{v}\varvec{e}}$ (kW)	$\:{\varvec{P}}_{\varvec{L},\varvec{m}\varvec{a}\varvec{x}}$ (kW)	$\:\varvec{S}\varvec{t}\varvec{d}$	$\:{\varvec{Q}}_{\varvec{L}}$ (kVar)	$\:\varvec{T}\varvec{V}\varvec{D}$ (pu)	$\:\varvec{V}\varvec{S}\varvec{I}$ (pu)	$\:{\varvec{V}}_{\varvec{L}}$ (pu)	$\:{\varvec{V}}_{\varvec{U}}$ (pu)	Time (min)
GWO	64.523	64.609	65.710	3.00E-01	41.871	1.428	0.1815	0.9508	1	4.89
JS	64.523	64.523	64.524	1.97E-04	41.536	1.422	0.1822	0.9506	1	5.20
AEO	64.523	64.523	64.523	2.12E-14	41.536	1.423	0.1822	0.9506	1	10.19
MVO	64.523	64.811	65.710	4.56E-01	42.188	1.436	0.1815	0.9508	1	5.01
FPA	64.526	64.706	65.203	1.50E-01	41.827	1.428	0.1824	0.9506	1	5.19
PSO	64.523	64.717	65.710	4.12E-01	42.129	1.432	0.1810	0.9509	1	5.40
GNDO	64.523	64.582	65.315	1.53E-01	41.666	1.428	0.1827	0.9505	1	10.03
DA	64.523	64.795	65.914	3.96E-01	42.023	1.435	0.1824	0.9505	1	15.72
BO	64.523	64.766	65.710	3.92E-01	42.054	1.437	0.1827	0.9505	1	6.11
SPO	64.523	64.921	65.710	4.77E-01	49.744	1.340	0.1792	0.9514	1.0097	21.09
JAYA	64.523	64.541	64.807	4.94E-02	51.245	1.682	0.2261	0.9371	1.0011	5.02
SSA	64.523	64.840	65.710	4.24E-01	42.210	1.442	0.1828	0.9504	1	5.07
ALO	64.523	65.014	65.914	4.99E-01	43.026	1.449	0.1794	0.9513	1	6.14
CGO	64.523	64.523	64.523	3.12E-08	72.133	2.165	0.2819	0.9194	1.0006	20.97
CStA	64.528	64.624	64.937	9.52E-02	48.270	1.533	0.1887	0.9483	1.0029	21.00
SMA	64.523	64.809	65.710	4.18E-01	75.126	2.091	0.2586	0.9264	1	5.15
HHO	64.523	65.076	67.202	7.07E-01	42.472	1.443	0.1829	0.9504	1	8.94
AOA	64.596	68.520	72.901	2.06E + 00	45.064	1.497	0.1898	0.9483	1	5.10
GOA	64.558	66.077	70.773	1.23E + 00	43.622	1.479	0.1832	0.9501	1	30.29
AOS	64.575	66.232	69.090	1.31E + 00	61.570	1.812	0.2349	0.9340	1.0031	5.44

Table 5 reveals several critical characteristics upon closer examination. The approaches tend to converge to virtually the same outcome in every application, except the AOA, GOA, and AOS algorithms, as indicated by the extremely low values of the standard deviation. The fastest algorithms are GWO, MVO, JAYA, SSA, and AOA, in that order. GOA, on the other hand, is the slowest method, with an execution time of 30.29 min, respectively. The upper voltage limit for all approaches remains constant at 1.0 pu, except for five algorithms that have slightly higher values. The lower voltage values are nearly identical, except for CGO and SMA, which exhibit lower values, as evidenced by the TVD values.

### Performance of the 69-bus system

**Fig. 13:**
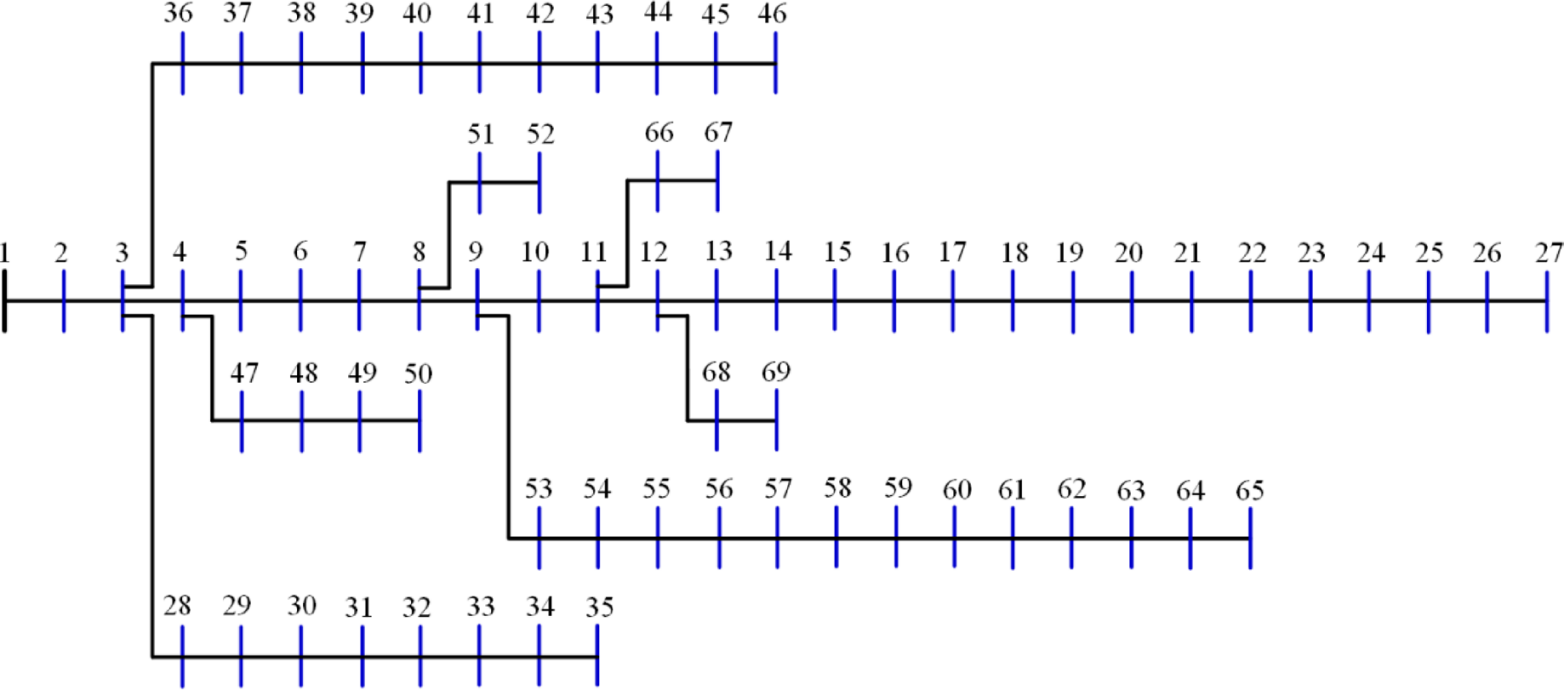
Layout of the 69-bus system.

**Fig. 14:**
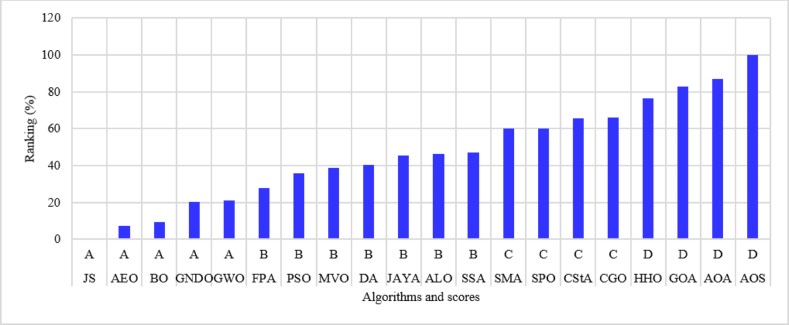
Ranking of algorithms and their scores with the 69-bus system.

The 12.66 kV 69-bus system has a total demand of $\:3801.89+j2694.10$ kVA^64^, as shown in Fig. 13. The implemented algorithms reduce power loss by efficiently allocating two DGs. The algorithms execute 50 times for statistical analysis; the performance metrics are detailed in Table 6. The percentage rankings and scores of the algorithms are calculated and listed in Fig. 14. It is noted that the JS, AEO, BO, GNDO, and GWO algorithms have achieved the highest scores of (A) The second position is allocated to the FPA, PSO, MVO, DA, JAYA, ALO, and SSA algorithms, each receiving a score of (B) The SMA, SPO, CStA, and CGO algorithms rank third with a score of (C) The fourth position is held by the lowest-ranked algorithms, HHO, GOA, AOA, and AOS, each receiving a score of D.

**Table 6:** Performance parameters of the 69-bus system with different algorithms.

Method	$\:{\varvec{P}}_{\varvec{L},\varvec{m}\varvec{i}\varvec{n}}$ (kW)	$\:{\varvec{P}}_{\varvec{L},\varvec{a}\varvec{v}\varvec{e}}$ (kW)	$\:{\varvec{P}}_{\varvec{L},\varvec{m}\varvec{a}\varvec{x}}$ (kW)	$\:\varvec{S}\varvec{t}\varvec{d}$	$\:{\varvec{Q}}_{\varvec{L}}$ (kVar)	$\:\varvec{T}\varvec{V}\varvec{D}$ (pu)	$\:\varvec{V}\varvec{S}\varvec{I}$ (pu)	$\:{\varvec{V}}_{\varvec{L}}$ (pu)	$\:{\varvec{V}}_{\varvec{U}}$ (pu)	Time (min)
JS	71.644	71.644	71.644	5.67E-14	35.928	0.499	0.082	0.9789	1	7.50
AEO	71.644	71.644	71.644	4.26E-14	35.928	0.499	0.082	0.9789	1	14.69
BO	71.644	71.888	74.463	6.31E-01	35.974	0.510	0.082	0.9789	1	7.48
GNDO	71.644	71.814	74.463	6.76E-01	35.970	0.506	0.082	0.9789	1	16.23
GWO	71.644	72.048	74.567	1.01E + 00	36.041	0.517	0.082	0.9788	1	8.32
FPA	71.741	72.459	73.936	4.63E-01	36.205	0.513	0.082	0.9789	1	8.54
PSO	71.644	72.010	78.961	1.49E + 00	36.053	0.511	0.084	0.9783	1	8.77
MVO	71.644	72.513	78.961	1.92E + 00	36.198	0.531	0.085	0.9779	1	7.92
DA	71.644	73.065	74.703	1.39E + 00	36.331	0.562	0.082	0.9788	1	15.20
JAYA	71.644	71.653	71.919	3.89E-02	61.151	0.787	0.169	0.9537	1.0056	7.84
ALO	71.644	72.927	78.053	1.52E + 00	36.272	0.557	0.083	0.9786	1	9.48
SSA	71.644	73.131	78.961	2.11E + 00	36.354	0.556	0.085	0.9780	1	7.04
SMA	71.644	72.100	78.961	1.76E + 00	56.684	0.913	0.158	0.9565	1	8.05
SPO	71.644	72.045	74.567	1.00E + 00	46.969	0.605	0.116	0.9692	1.0045	32.19
CStA	71.659	72.573	74.862	7.44E-01	41.301	0.643	0.101	0.9734	1.0011	32.00
CGO	71.644	71.700	74.463	3.99E-01	85.488	1.408	0.269	0.9240	1.0072	38.72
HHO	71.651	74.271	83.166	2.81E + 00	36.767	0.589	0.089	0.9770	1	13.39
GOA	71.936	78.585	84.625	3.82E + 00	38.485	0.668	0.104	0.9728	1.0001	17.57
AOA	73.654	84.381	102.160	6.40E + 00	41.098	0.725	0.113	0.9705	1.0007	9.23
AOS	72.193	79.061	87.059	4.02E + 00	61.056	0.918	0.183	0.9497	1.0036	9.17

After inspecting Table 6 for the 69-bus system, it is observed that the power losses vary among some algorithms, reflected in other parameters such as TVD and VSI. The AOA algorithm has the highest power loss of 84.38 kW, while many other algorithms reach only 71.64 kW. The upper voltage remains constant at 1.0 pu except for seven algorithms. The lower voltages are above 0.95 pu except for the CGO and AOS methods. Some algorithms, like SSA, BO, and JS, show fast convergence; others, such as CGO, SPO, and CStA, are very slow.

### Performance of the 85-bus system

**Fig. 15:**
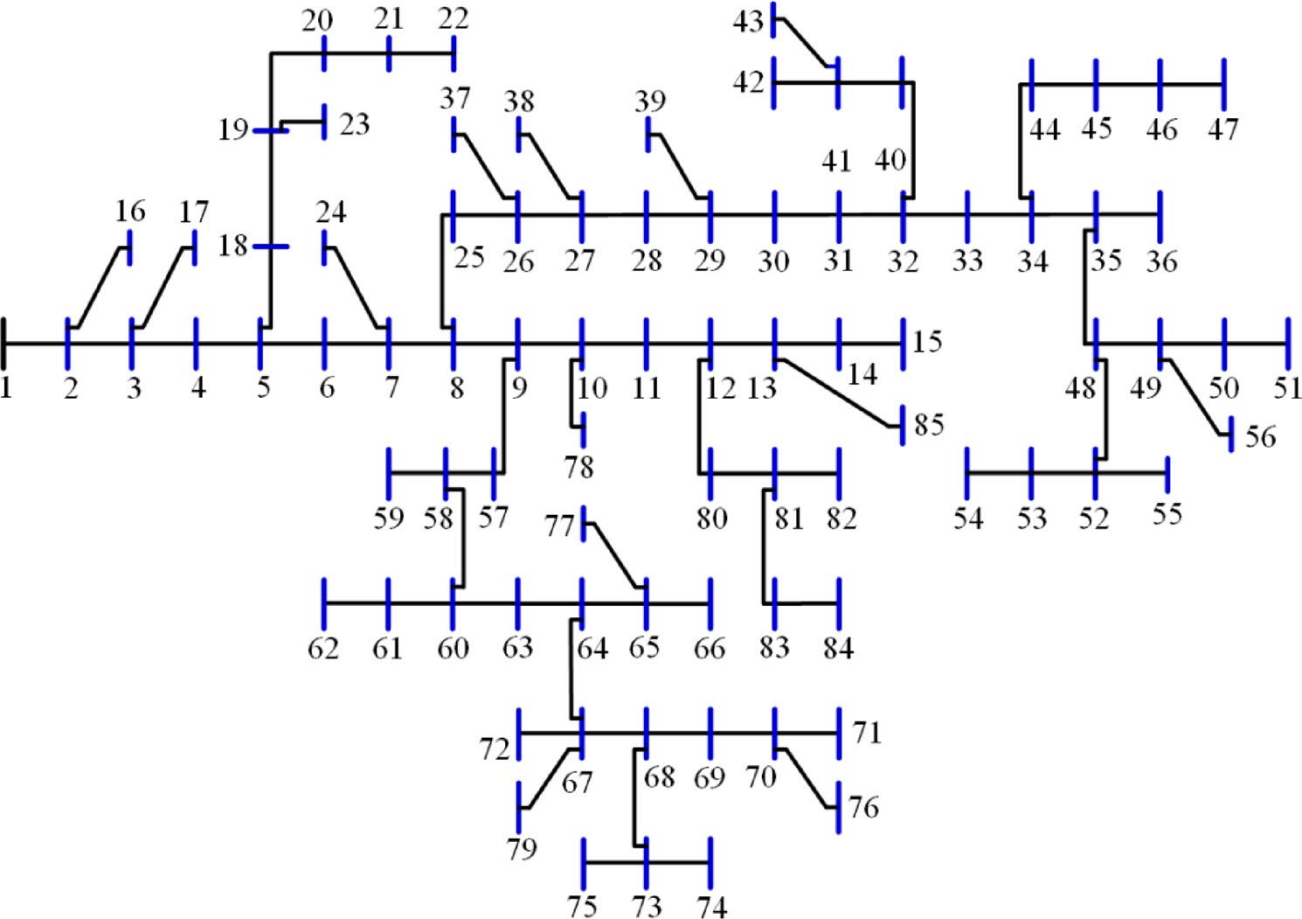
Layout of the 85-bus system.

**Fig. 16:**
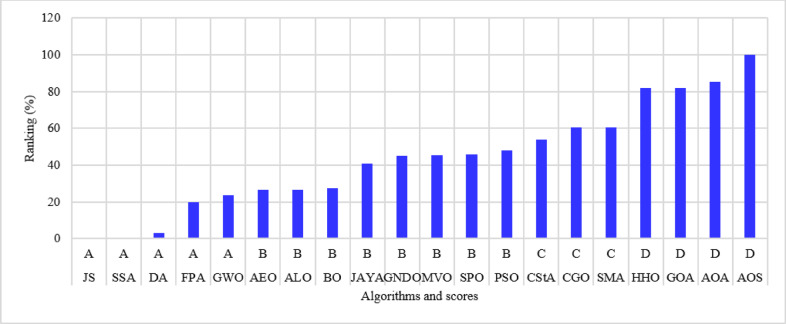
Ranking of algorithms and their scores with the 85-bus system.

The 11 kV 85-bus system has a total demand of $\:2570.28+j2622.08$ kVA^65^, as shown in Fig. 15. The system consists of many interconnected sub-branches. As in previous cases, the system is optimized by allocating two DGs using different algorithms to minimize system loss. The performance metrics from the 50 runs are documented in Table 7, and the ranking with scores is shown in Fig. 16. With this system, five algorithms achieve the A score: JS, SSA, DA, FPA, and GWO. The most effective algorithms are JS and SSA, whilst the least effective is AOS. Eight algorithms received a score of B, three attained a score of C, and four received the lowest score of D.

**Table 7:** Performance parameters of the 85-bus system with different algorithms.

Method	$\:{\varvec{P}}_{\varvec{L},\varvec{m}\varvec{i}\varvec{n}}$ (kW)	$\:{\varvec{P}}_{\varvec{L},\varvec{a}\varvec{v}\varvec{e}}$ (kW)	$\:{\varvec{P}}_{\varvec{L},\varvec{m}\varvec{a}\varvec{x}}$ (kW)	$\:\varvec{S}\varvec{t}\varvec{d}$	$\:{\varvec{Q}}_{\varvec{L}}$ (kVar)	$\:\varvec{T}\varvec{V}\varvec{D}$ (pu)	$\:\varvec{V}\varvec{S}\varvec{I}$ (pu)	$\:{\varvec{V}}_{\varvec{L}}$ (pu)	$\:{\varvec{V}}_{\varvec{U}}$ (pu)	Time (min)
JS	156.40	156.40	156.40	3.14E-04	95.837	3.157	0.205	0.9442	1	7.14
SSA	156.40	156.40	156.40	1.35E-13	95.836	3.157	0.205	0.9442	1	9.97
DA	156.40	156.58	157.01	2.24E-01	95.941	3.109	0.203	0.9448	1	14.57
FPA	156.40	156.90	157.73	3.65E-01	96.087	3.168	0.205	0.9441	1	8.92
GWO	156.40	157.11	165.27	2.43E + 00	96.205	3.196	0.208	0.9434	1	6.88
AEO	156.40	156.67	158.79	4.86E-01	95.981	3.169	0.202	0.9450	1	17.44
ALO	156.40	157.64	159.84	9.73E-01	96.641	3.218	0.197	0.9465	1	8.44
BO	156.40	157.34	158.79	1.03E + 00	96.506	3.213	0.199	0.9461	1	10.92
JAYA	156.40	156.40	156.41	2.37E-03	116.660	4.308	0.262	0.9262	1.0002	6.98
GNDO	156.40	157.00	165.27	2.00E + 00	96.131	3.196	0.208	0.9434	1	18.50
MVO	156.40	157.64	165.27	3.11E + 00	96.481	3.225	0.210	0.9428	1	7.45
SPO	156.40	157.75	165.27	2.89E + 00	108.800	2.914	0.202	0.9452	1.0004	38.02
PSO	156.40	157.43	165.27	2.84E + 00	96.368	3.210	0.209	0.9431	1	9.55
CStA	156.40	156.52	156.89	1.13E-01	103.250	3.573	0.228	0.9369	1	29.76
CGO	156.40	156.40	156.40	1.07E-13	143.600	5.819	0.339	0.9008	1.0003	29.54
SMA	156.40	156.58	165.27	1.25E + 00	137.040	5.104	0.300	0.9127	1	7.15
HHO	156.40	159.27	170.49	4.40E + 00	97.205	3.263	0.213	0.9417	1	13.45
GOA	156.40	163.34	174.27	5.50E + 00	98.996	3.341	0.217	0.9406	1	15.08
AOA	159.50	166.89	176.12	3.75E + 00	101.690	3.628	0.224	0.9382	1	7.06
AOS	157.38	162.49	170.45	3.46E + 00	123.100	4.124	0.263	0.9256	1.0007	7.50

Upon examining Table 7 for the 85-bus system, it is noted that power losses differ across various algorithms, as indicated by other characteristics such as TVD and VSI. The AOA algorithm exhibits the most significant power loss at 166.89 kW, whilst numerous other algorithms attain a maximum of just 156.40 kW. The top voltage remains fixed at 1.0 pu, except for four methods. The lower voltages exceed 0.93 pu, except for the JAYA, CGO, SMA, and AOS techniques. The CGO attains the lowest minimum voltage of 0.9008 pu, while ALO reaches the greatest voltage of 0.9465 pu. Certain algorithms, such as GWO, JAYA, and AOA, demonstrate rapid convergence, but others, like SPO, CGO, and CStA, have significantly slower convergence rates.

### Performance of the 94-bus system

**Fig. 17:**
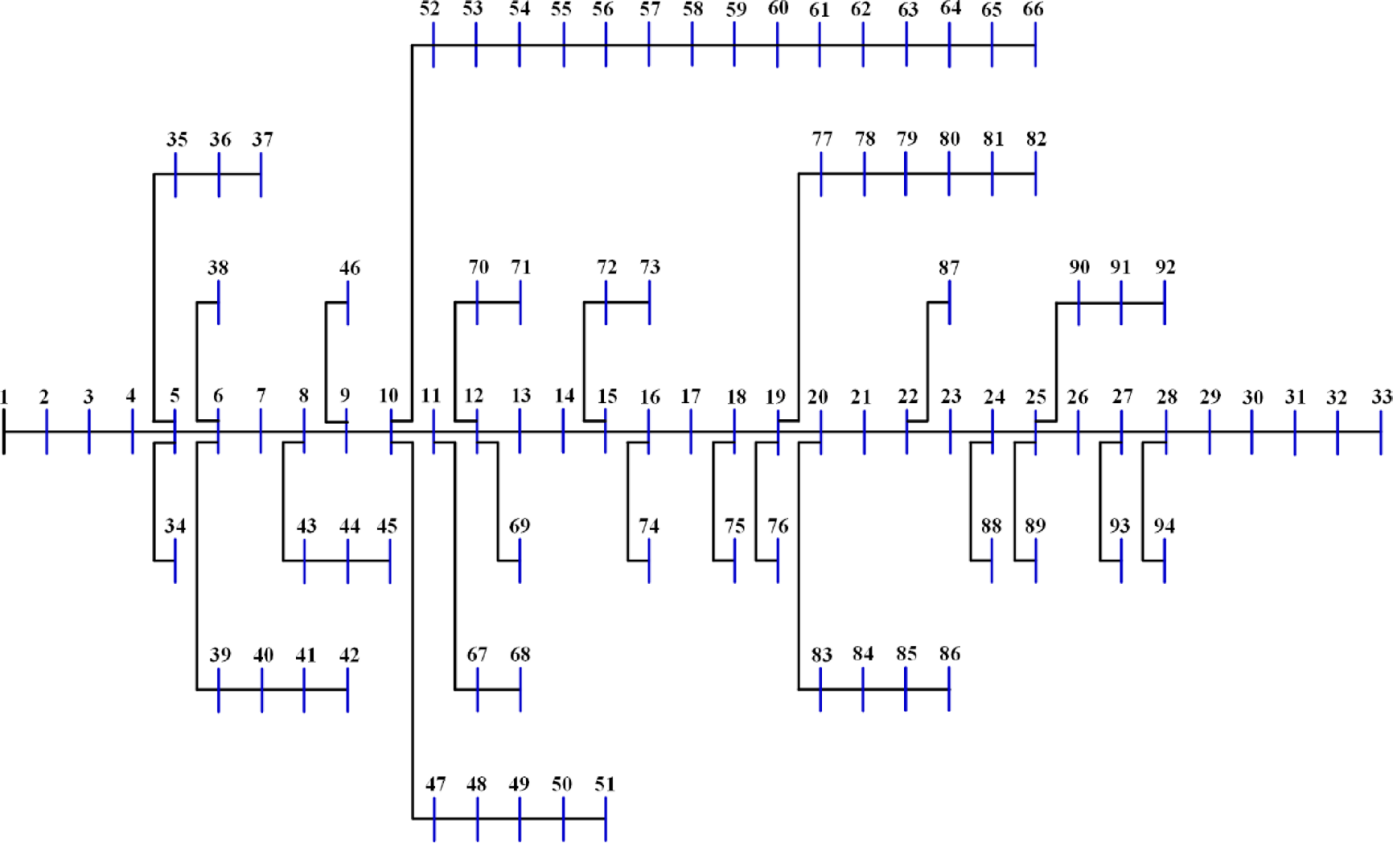
Layout of the 94-bus system.

**Fig. 18:**
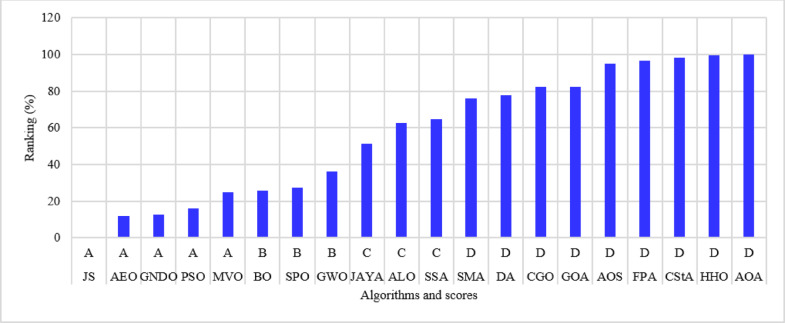
Ranking of algorithms and their scores with the 94-bus system.

The 94-bus system has a total demand of $\:4797+j2323.9$ kVA with a rated voltage of 15 kV^66^, shown in Fig. 17. The system has many branches and is optimized by allocating four DGs distributed optimally among the system buses. The system performance with different algorithms is listed in Table 8. The ranking of the algorithms and their scores is shown in Fig. 18. Five algorithms, JS, AEO, GNDO, PSO, and MVO, have attained a first rank of under 25% with a score of (A) Three algorithms, BO, SPO, and GWO, have attained a second-place rating with a score of (B) Three other algorithms were assigned a score of C: JAYA, ALO, and SSA. The algorithms with the lowest rankings include SMA, DA, CGO, GOA, AOS, FPA, CStA, HHO, and AOA, all of which received a score of D.

**Table 8:** Performance parameters of the 94-bus system with different algorithms.

Method	$\:{P}_{L,min}$ (kW)	$\:{P}_{L,ave}$ (kW)	$\:{P}_{L,max}$ (kW)	$\:Std$	$\:{Q}_{L}$ (kVar)	$\:TVD$ (pu)	$\:VSI$ (pu)	$\:{V}_{L}$ (pu)	$\:{V}_{U}$ (pu)	Time (min)
JS	68.17	68.17	68.20	0.008	88.95	3.46	0.204	0.9446	1	6.82
AEO	68.17	68.19	68.59	0.092	88.94	3.46	0.204	0.9445	1	16.03
GNDO	68.17	68.23	69.28	0.191	88.99	3.46	0.204	0.9445	1	12.77
PSO	68.17	68.36	72.46	0.617	88.98	3.47	0.204	0.9444	1	7.16
MVO	68.17	68.78	72.49	1.276	89.11	3.48	0.206	0.9438	1	6.78
BO	68.17	68.62	72.49	1.176	89.03	3.47	0.206	0.9439	1	6.27
SPO	68.17	68.28	72.46	0.634	141.78	3.03	0.203	0.9448	1.0148	29.80
GWO	68.17	69.09	72.98	1.493	89.31	3.49	0.207	0.9437	1	6.96
JAYA	68.20	68.44	69.02	0.195	94.64	3.54	0.210	0.9426	1	6.99
ALO	68.17	70.04	75.50	1.864	89.95	3.49	0.207	0.9435	1	9.67
SSA	68.17	70.13	74.50	1.778	90.06	3.51	0.208	0.9433	1	6.91
SMA	68.17	69.57	72.49	1.929	277.89	6.23	0.343	0.8971	1	6.93
DA	68.31	70.50	74.95	1.726	90.21	3.51	0.211	0.9423	1	20.23
CGO	68.17	68.57	72.46	1.168	205.99	5.56	0.316	0.9082	1.0037	31.21
GOA	68.75	73.05	79.58	2.425	92.23	3.51	0.205	0.9441	1	25.15
AOS	68.22	69.70	73.14	1.405	135.41	3.78	0.233	0.9350	1.003	8.72
FPA	70.98	73.95	76.68	1.609	93.46	3.61	0.218	0.9404	1	7.49
CStA	68.91	71.09	73.85	1.096	106.81	3.84	0.229	0.9369	1	30.84
HHO	68.47	72.96	86.89	3.410	92.11	3.54	0.209	0.9430	1	11.92
AOA	70.71	76.00	90.56	3.537	94.91	3.48	0.216	0.9410	1	9.35

Optimizing the 94-bus system with 20 algorithms and evaluating the performance metrics from 50 runs, as shown in Table 8, reveals different characteristics and observations. It is observed that power losses vary among the algorithms, as reflected by metrics like TVD and VSI. The AOA algorithm shows the highest power loss at 76.0 kW, while many other algorithms have a maximum just below 68.5 kW. The top voltage remains steady at 1.0 pu, except for three methods (SPO, CGO, and AOS). Most methods maintain lower voltages above 0.93 pu. The SMA records the lowest minimum voltage at 0.8971 pu, while SPO reaches the highest voltage at 0.9448 pu. Certain algorithms, such as BO, show rapid convergence, whereas others, like SPO, CGO, and CStA, exhibit significantly slower convergence rates.

### Performance of the 108-bus system

**Fig. 19:**
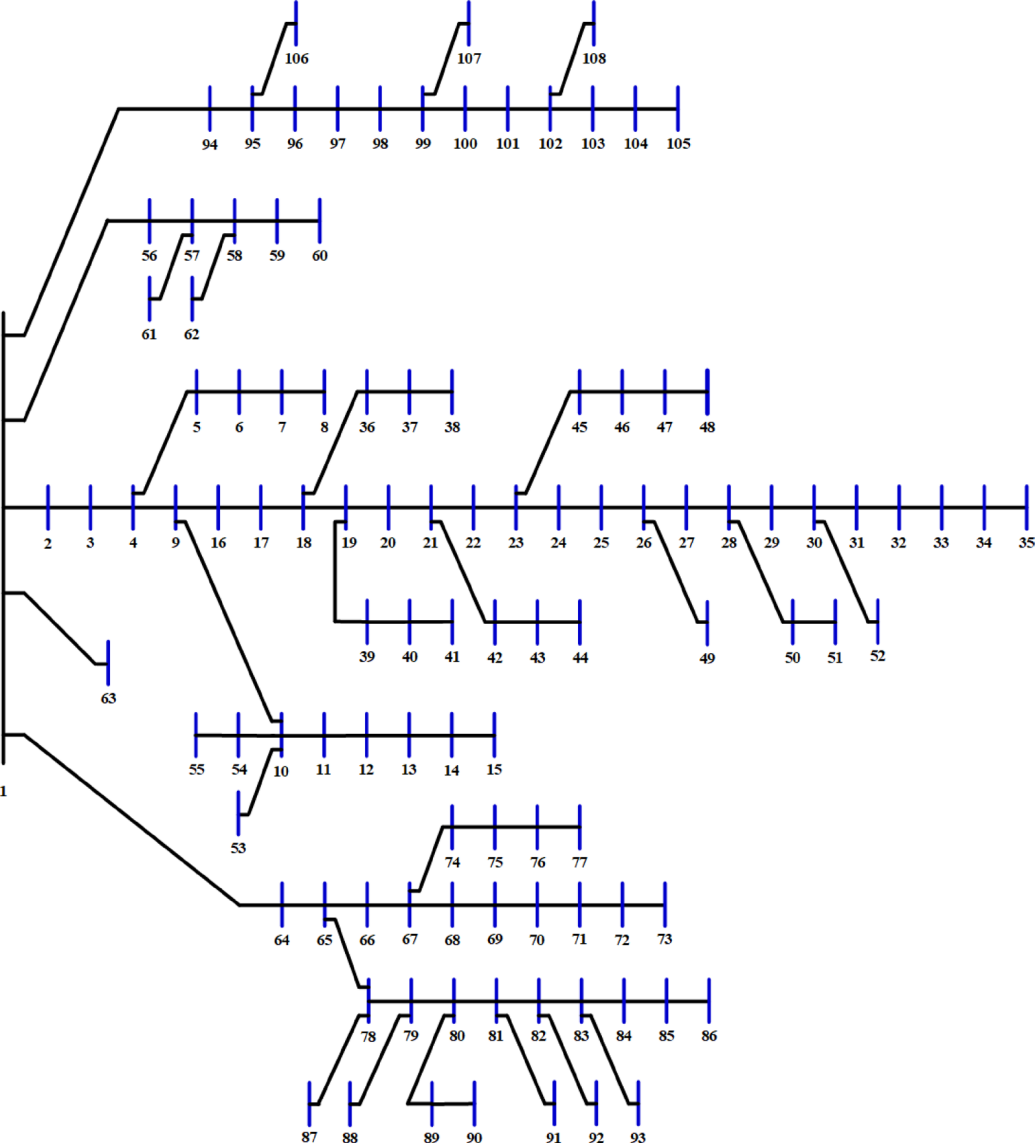
Layout of the 108-bus system.

**Fig. 20:**
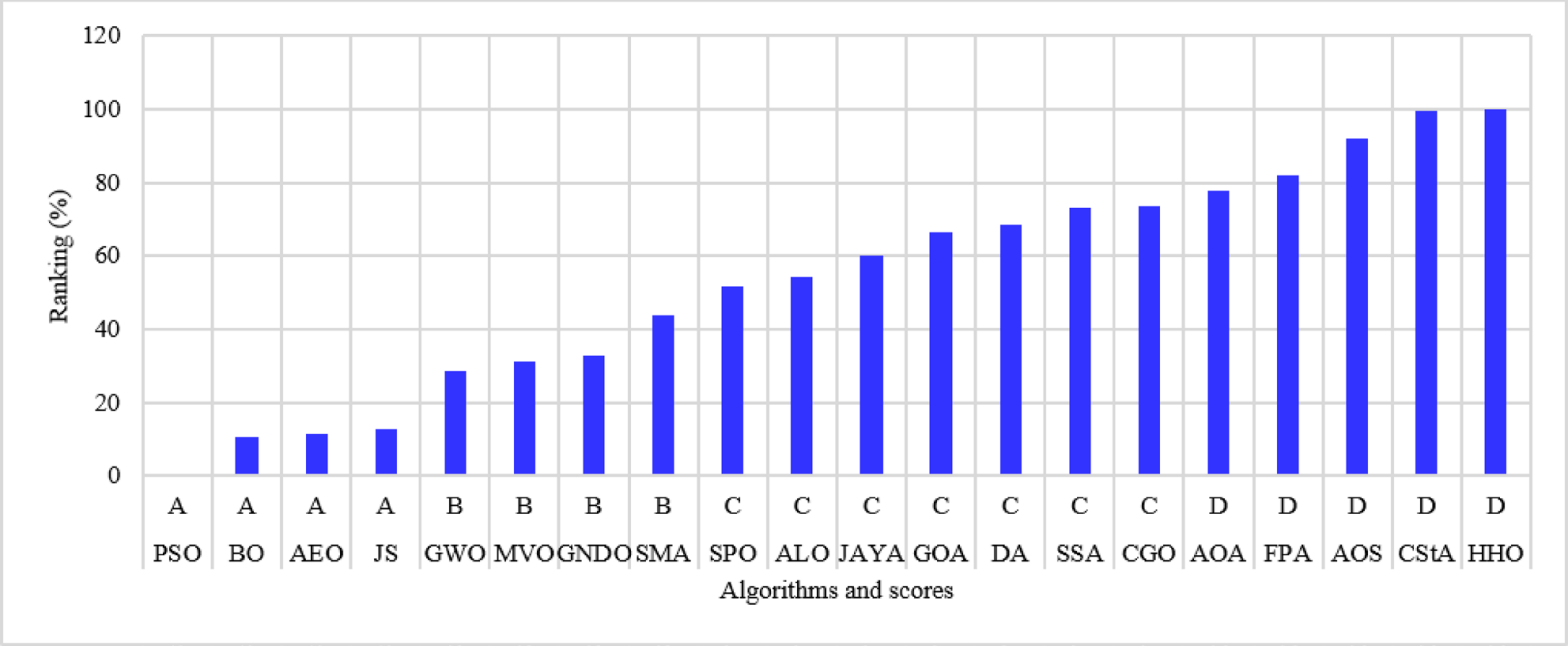
Ranking of algorithms and their scores with the 108-bus system.

The 108-bus system is shown in Fig. 19 and has a total demand of 12,132 + j9099 kVA with a rating voltage of 11 kV^67^. The system comprises numerous branches linked to the substation bus. The system is optimized by strategically assigning four DGs utilizing the 20 algorithms at ideal locations and sizes. Each algorithm is executed 50 times for statistical analysis, and the system behavior is presented in Table 9. The percentage ranking and scores are calculated and shown in Fig. 20. The algorithms PSO, BO, AEO, and JS have attained the highest ranking, receiving a score of A. B-score algorithms occupy the second position, including GWO, MVO, GNDO, and SMA. The third position is allocated to the SPO, ALO, JAYA, GOA, DA, SSA, and CGO algorithms, with a score of C. The algorithms with the lowest rankings are AOA, FPA, AOS, CstA, and HHO, with a score of D and ascending order.

**Table 9:** Performance parameters of the 108-bus system with different algorithms.

Method	$\:{P}_{L,min}$ (kW)	$\:{P}_{L,ave}$ (kW)	$\:{P}_{L,max}$ (kW)	$\:Std$	$\:{Q}_{L}$ (kVar)	$\:TVD$ (pu)	$\:VSI$ (pu)	$\:{V}_{L}$ (pu)	$\:{V}_{U}$ (pu)	Time (min)
PSO	306.73	309.80	311.95	1.26	156.32	2.30	0.145	0.9605	1	9.69
BO	306.73	310.21	312.39	1.38	156.45	2.30	0.145	0.9605	1	9.89
AEO	306.73	308.19	311.28	1.63	155.73	2.28	0.145	0.9605	1	17.64
JS	306.77	310.41	312.38	1.02	156.78	2.31	0.145	0.9605	1	8.33
GWO	309.42	317.35	341.41	8.37	161.14	2.36	0.145	0.9605	1.0001	9.01
MVO	310.33	316.17	325.30	3.57	160.09	2.33	0.146	0.9604	1.0001	7.96
GNDO	306.73	313.17	356.00	9.09	158.49	2.32	0.145	0.9605	1	15.47
SMA	306.73	309.64	311.28	1.29	221.18	2.86	0.212	0.9396	1.0092	9.80
SPO	306.73	309.81	312.39	1.37	191.16	2.34	0.166	0.9539	1.0277	33.42
ALO	309.63	321.65	361.92	12.99	164.00	2.30	0.146	0.9604	1.0002	10.35
JAYA	309.53	313.54	322.72	2.67	197.22	2.36	0.185	0.9484	1.0079	8.84
GOA	311.90	335.70	381.52	19.74	158.49	2.32	0.145	0.9605	1	25.57
DA	309.63	324.37	363.04	15.20	167.89	2.35	0.147	0.9602	1	23.18
SSA	313.81	331.88	368.41	14.22	173.10	2.41	0.146	0.9605	1.0002	9.64
CGO	306.73	310.25	311.95	1.39	267.02	2.93	0.321	0.9055	1.0151	33.42
AOA	326.71	373.50	395.70	14.47	189.37	2.15	0.158	0.9568	1.0104	8.74
FPA	315.08	333.36	355.91	9.42	169.01	2.42	0.158	0.9570	1.0016	11.02
AOS	309.58	321.46	341.63	7.11	236.56	2.44	0.232	0.9332	1.0364	10.58
CStA	316.43	334.23	357.98	9.17	206.25	2.38	0.179	0.9503	1.0068	42.97
HHO	311.64	344.77	388.34	21.08	180.65	2.38	0.152	0.9584	1.0008	15.95

Optimizing the 108-bus system using 20 algorithms and evaluating performance metrics over 50 runs, as shown in Table 9, reveals various features and insights. Power losses vary among the algorithms, indicated by measures such as TVD and VSI. The AOA algorithm exhibits the highest power loss at 373.5 kW, whereas many other algorithms have a power loss of approximately 310.0 kW. The maximum voltage remains above 1.0 pu for most of the algorithms, but the value doesn’t exceed 1.05 pu. Most methods maintain voltages above 0.96 pu. The CGO records the lowest lower voltage at 0.9055 pu. Some algorithms, such as MVO, exhibit rapid convergence, whereas others, including SPO, CGO, and CStA, demonstrate noticeably slower convergence rates.

### Performance of the 118-bus system

**Fig. 21:**
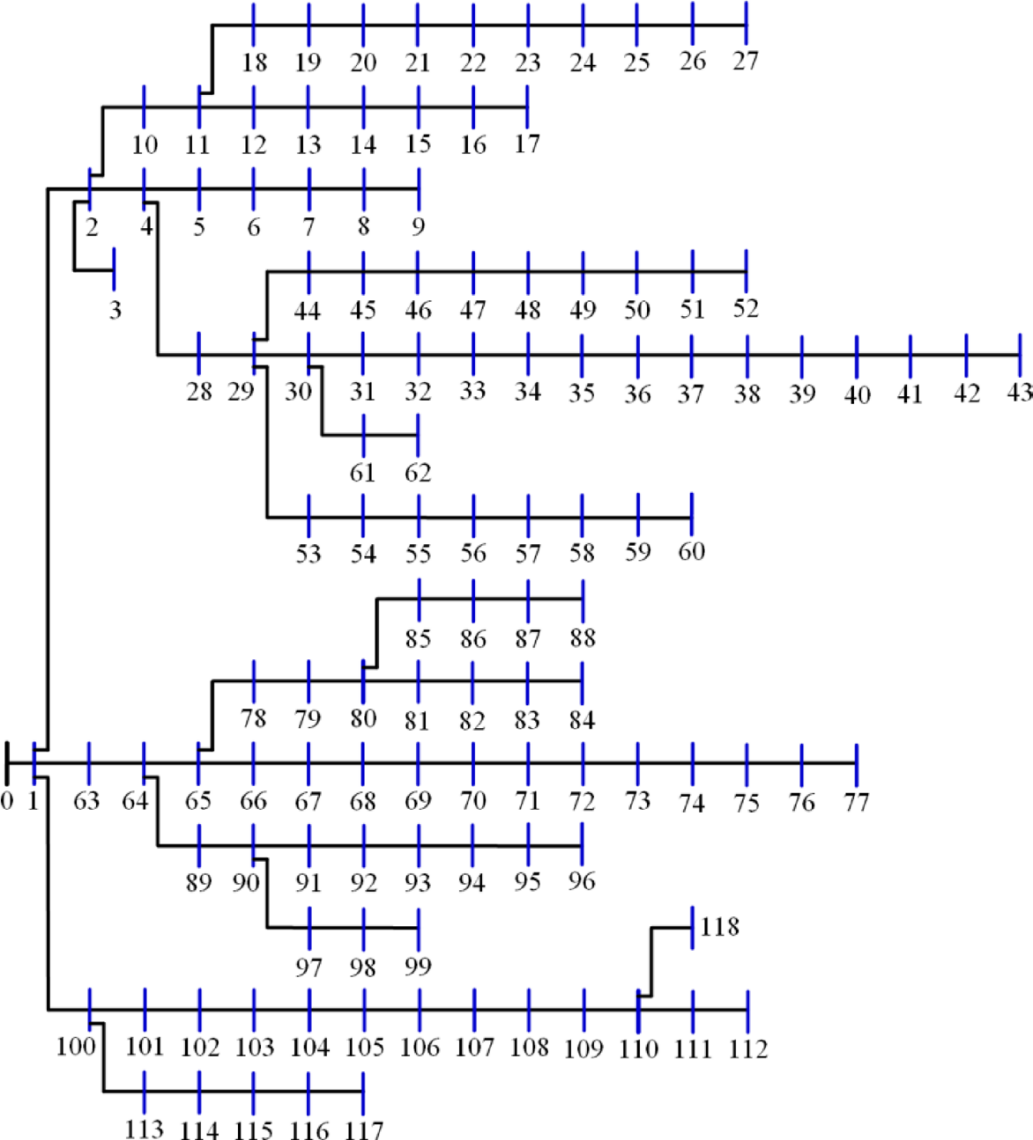
Layout of the 118-bus system.

**Fig. 22:**
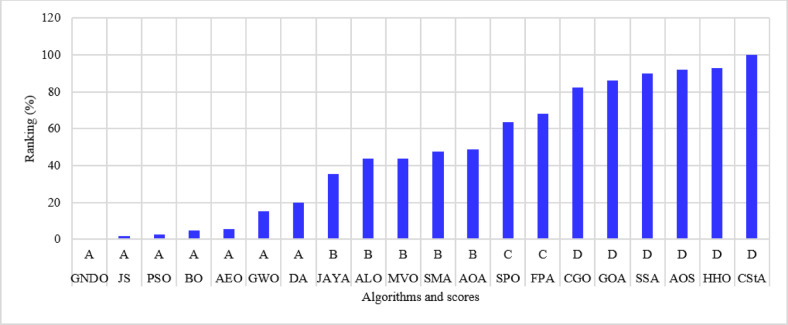
Ranking of algorithms and their scores with the 118-bus system.

The 118-bus system has many primary and secondary branches, as shown in Fig. 21. The system has a total demand of $\:22709.720+j17041.068$ kVA and a rating voltage of 11 kV^68^. As a large-scale system, it is optimized by allocating four DGs. The system is simulated using the 20 algorithms, and the performance metrics are listed in Table 10. The percentage ranking and scores of the algorithms are shown in Fig. 22. Seven algorithms achieved a score below 25% of the A score. The GNDO is the most effective algorithm, while the CStA is the least effective. Seven algorithms, GNDO, JS, PSO, BO, AEO, GWO, and DA, are ranked first with an overall rating of A. Five algorithms are ranked second with a score of B, whereas two are ranked third with a score of C. The six lowest-ranked algorithms each received a score of D.

**Table 10:** Performance parameters of the 118-bus system with different algorithms.

Method	$\:{P}_{L,min}$ (kW)	$\:{P}_{L,ave}$ (kW)	$\:{P}_{L,max}$ (kW)	$\:Std$	$\:{Q}_{L}$ (kVar)	$\:TVD$ (pu)	$\:VSI$ (pu)	$\:{V}_{L}$ (pu)	$\:{V}_{U}$ (pu)	Time (min)
GNDO	616.16	617.12	618.88	0.67	470.05	2.82	0.167	0.9541	1	21.00
JS	616.16	616.30	617.05	0.18	469.50	2.82	0.167	0.9540	1	13.50
PSO	616.16	616.51	617.89	0.65	468.33	2.83	0.167	0.9541	1	27.04
BO	616.16	617.86	623.69	2.33	467.72	2.86	0.168	0.9541	1	12.60
AEO	616.16	616.48	618.72	0.64	468.85	2.83	0.167	0.9541	1	23.62
GWO	616.57	619.68	627.10	2.62	470.64	2.86	0.168	0.9540	1	10.93
DA	616.17	622.33	646.43	5.10	468.28	2.85	0.167	0.9547	1	19.90
JAYA	616.16	617.32	620.43	1.13	507.15	3.09	0.223	0.9373	1.0003	12.15
ALO	616.33	621.39	633.98	4.33	470.32	2.88	0.173	0.9526	1	13.75
MVO	617.13	624.96	646.54	4.85	472.09	2.91	0.173	0.9529	1	12.73
SMA	616.16	618.34	627.74	3.39	584.36	3.38	0.234	0.9327	1	11.31
AOA	633.95	643.95	695.23	9.79	466.65	2.80	0.179	0.9513	1	10.83
SPO	616.16	617.75	627.74	2.69	576.25	3.23	0.252	0.9267	1.0098	44.24
FPA	626.56	639.32	658.69	7.79	480.93	2.98	0.194	0.9467	1	12.30
CGO	616.16	618.33	627.74	3.42	791.02	4.44	0.384	0.8850	1.0056	53.93
GOA	617.12	641.42	743.76	29.71	482.38	2.95	0.199	0.9448	1	26.13
SSA	622.35	648.70	695.78	15.16	505.11	3.24	0.205	0.9430	1	13.20
AOS	616.88	622.97	635.10	3.93	680.20	3.71	0.340	0.8998	1.0086	13.38
HHO	618.57	646.29	694.91	20.79	494.86	3.10	0.197	0.9457	1	19.14
CStA	624.35	636.09	650.28	5.76	573.56	3.37	0.263	0.9254	1.001	52.18

The 118-bus system has more branches than previous systems. Optimizing the 118-bus system using 20 algorithms and evaluating performance metrics over 50 runs, as listed in Table 10, reveals various features and insights. Power losses vary among the algorithms and are reflected in measures such as TVD and VSI. Most of the standard deviation of power loss is greater than unity and may reach 29.71 for the case of the GOA algorithm. The SSA algorithm exhibits the highest power loss at 648.70 kW, whereas many other algorithms have a power loss of approximately 620.0 kW. The maximum voltage remains at 1.0 pu for most of the algorithms; some voltages exceed 1.0 pu but are still under 1.05 pu. Most methods maintain voltages above 0.95 pu for the lower voltage value. The CGO records the lowest lower voltage at 0.8850 pu. Some algorithms, such as GWO, exhibit rapid convergence, whereas others, including SPO, CGO, and CStA, demonstrate noticeably slower convergence rates.

### 10. Performance of the 136-bus system

**Fig. 23:**
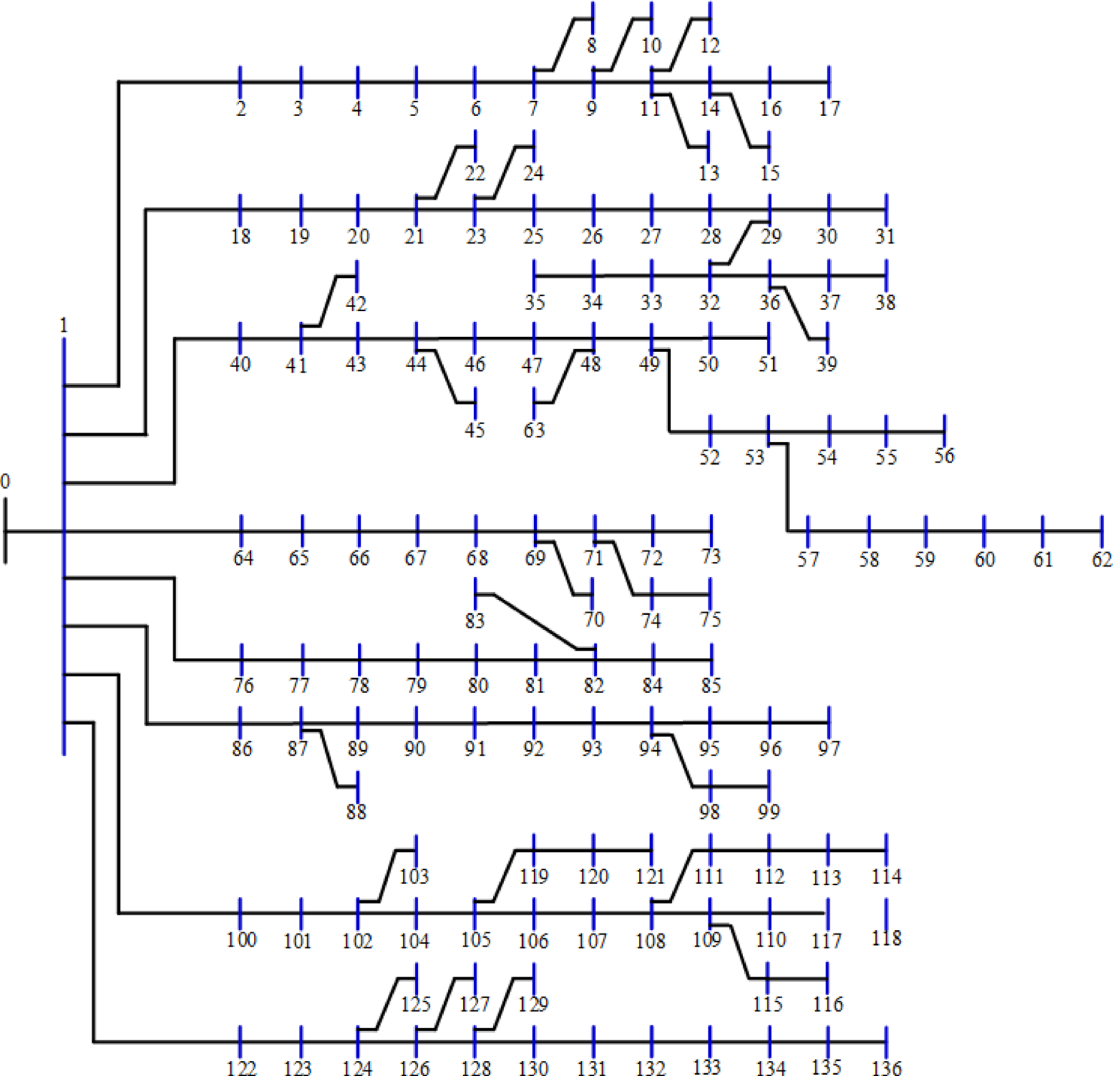
Layout of the 136-bus system.

**Fig. 24:**
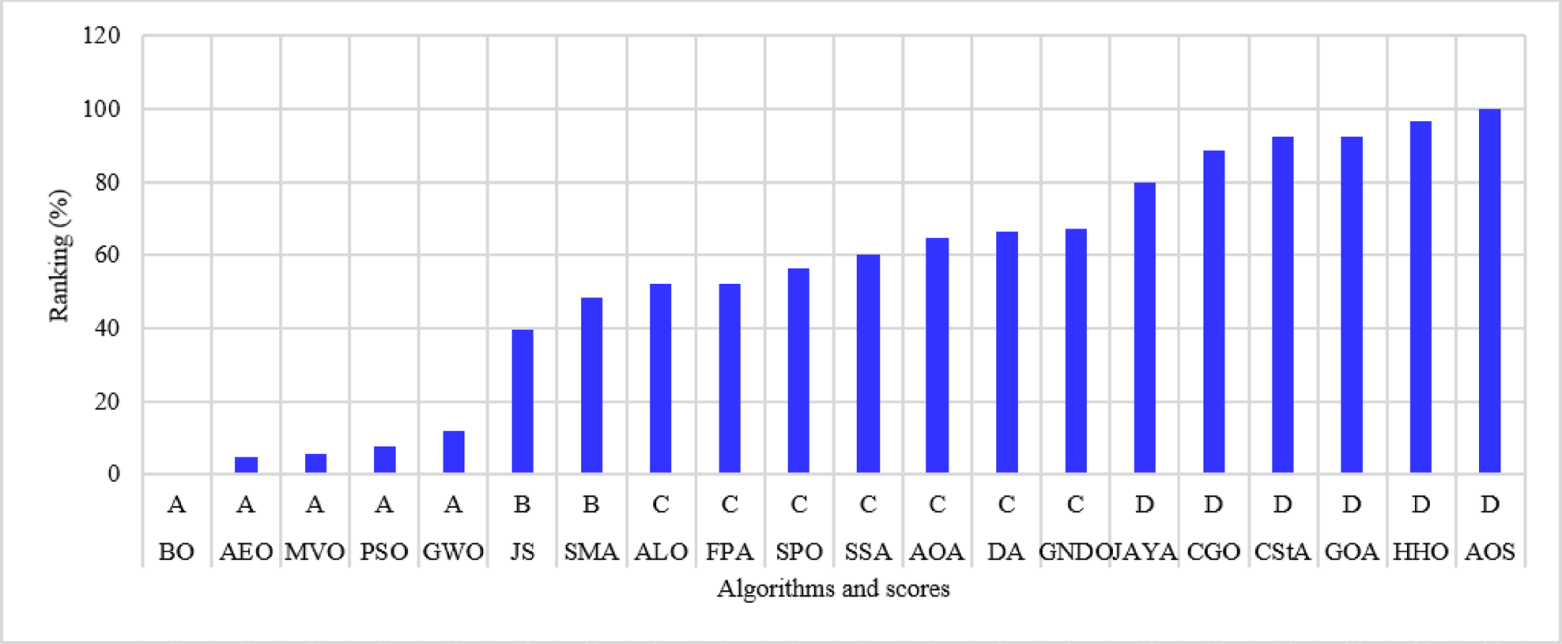
Ranking of algorithms and their scores with the 136-bus system.

The 136-bus system has eight main branches and other sub-branches, as shown in Fig. 23. The system demand is $\:18314.177+\:j7932.533$ kVA with a rated voltage of 13.8 kV^69^. The most extensive 136-bus system is optimized by four distributed generators utilizing the selected 20 algorithms. Each method is executed 50 times for statistical validity, addressing the stochastic characteristics of optimization algorithms. Table 11 presents the system performance. Figure 24 represents the ranking and scores of the 20 methods. Five algorithms, BO, AEO, MVO, PSO, and GWO, have attained the highest rating of below 25% with a score of A. The JS and SMA algorithms secured a score of B, placing them in second position. The algorithms ALO, FPA, SPO, SSA, AOA, DA, and GNDO are ranked third, but the algorithms JAYA, CGO, CStA, and GOA HHO and AOS are ranked lowest with a score of D.

**Table 11:** Performance parameters of the 136-bus system with different algorithms.

Method	$\:{P}_{L,min}$ (kW)	$\:{P}_{L,ave}$ (kW)	$\:{P}_{L,max}$ (kW)	$\:Std$	$\:{Q}_{L}$ (kVar)	$\:TVD$ (pu)	$\:VSI$ (pu)	$\:{V}_{L}$ (pu)	$\:{V}_{U}$ (pu)	Time (min)
BO	143.61	143.68	146.99	0.48	299.96	2.21	0.14	0.9637	1	10.86
AEO	143.61	143.62	143.92	0.04	299.81	2.21	0.14	0.9637	1	20.13
MVO	143.61	145.06	149.83	2.01	303.14	2.24	0.14	0.9635	1	9.11
PSO	143.61	144.61	149.79	1.77	302.17	2.23	0.14	0.9637	1	9.82
GWO	143.62	146.21	152.17	2.36	305.85	2.26	0.14	0.9638	1	9.37
JS	143.87	147.41	149.51	1.18	307.72	2.27	0.14	0.9625	1	10.70
SMA	143.61	144.06	148.62	1.23	450.14	2.63	0.18	0.9514	1.0001	11.04
ALO	143.72	147.75	157.69	3.11	308.73	2.27	0.14	0.9629	1	12.68
FPA	148.52	153.11	159.62	2.38	318.00	2.27	0.14	0.9638	1	10.59
SPO	143.61	143.92	149.79	1.01	434.34	2.35	0.18	0.9524	1.0047	37.93
SSA	143.92	149.45	160.25	3.37	312.57	2.31	0.14	0.9623	1	9.12
AOA	159.40	169.50	188.72	7.07	345.70	2.25	0.13	0.9650	1	10.55
DA	143.66	150.21	160.30	3.79	313.93	2.27	0.14	0.9623	1	21.26
GNDO	143.84	149.12	154.47	2.87	311.23	2.33	0.14	0.9618	1	19.66
JAYA	146.68	150.00	153.73	1.85	482.28	2.62	0.19	0.9495	1.0044	9.25
CGO	143.61	145.52	152.81	2.71	616.47	2.78	0.23	0.9353	1.0058	47.25
CStA	147.11	151.56	158.56	2.32	368.27	2.42	0.16	0.9585	1.0003	42.77
GOA	145.45	156.14	173.49	7.48	325.35	2.32	0.14	0.9624	1	29.30
HHO	144.17	152.37	170.72	5.57	318.90	2.31	0.15	0.9603	1	15.93
AOS	145.21	150.18	156.87	2.52	464.89	2.51	0.19	0.9487	1.0029	11.26

The 136-bus system possesses the largest branches among all systems. The optimization of the 136-bus system utilizing 20 algorithms and assessing performance metrics throughout 50 runs, as detailed in Table 11, uncovers diverse characteristics and insights. Power losses differ across algorithms and are reflected by metrics such as TVD and VSI. The majority of the standard deviation of power loss exceeds one and may attain a value of 7.48 in the case of the GOA algorithm. The AOA method demonstrates the most significant power loss at 169.5 kW, while numerous other algorithms exhibit a power loss of around 145.0 kW. The maximum voltage is consistently 1.0 pu for the majority of algorithms; however, certain values exceed 1.0 pu but remain below 1.05 pu. Most methods sustain voltages over 0.96 pu for the lower voltage threshold. The CGO registers the minimum lower voltage as 0.9353 pu. Certain algorithms, such as MVO, exhibit rapid convergence, whereas others, including SPO, CGO, and CStA, have significantly slower convergence rates.

## Overall ranking of optimization algorithms

**Fig. 25:**
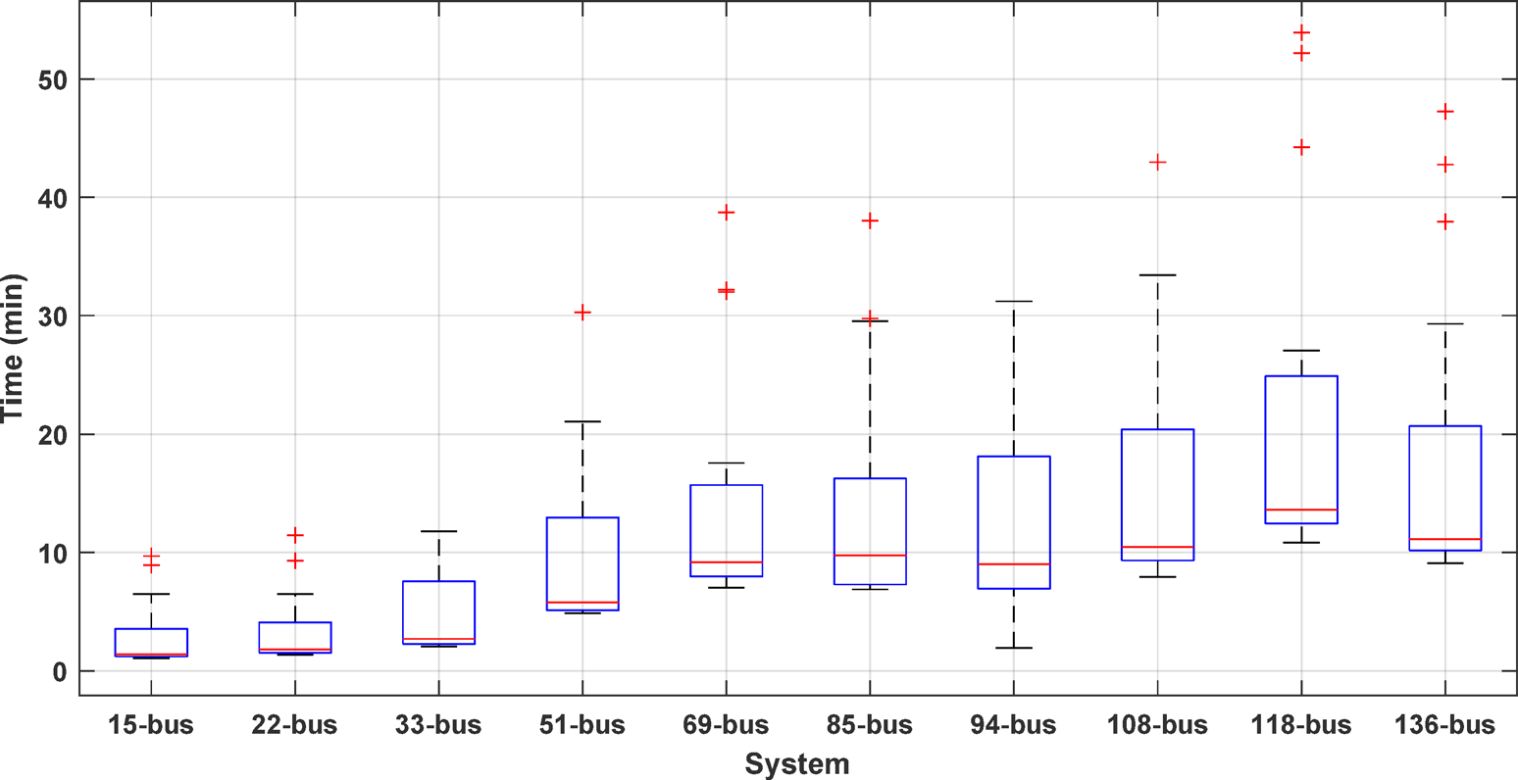
Execution time of the distribution systems solved by all optimization algorithms.

The execution times of the 20 algorithms for each studied distribution system are gathered and displayed in the box plot of Fig. 25. Increasing the system size has the effect of increasing the solution time of the optimization, and this increase is in proportion to the system size. Some systems have some outliers, indicating the different behaviors of the algorithms in solving the same distribution system.

**Fig. 26:**
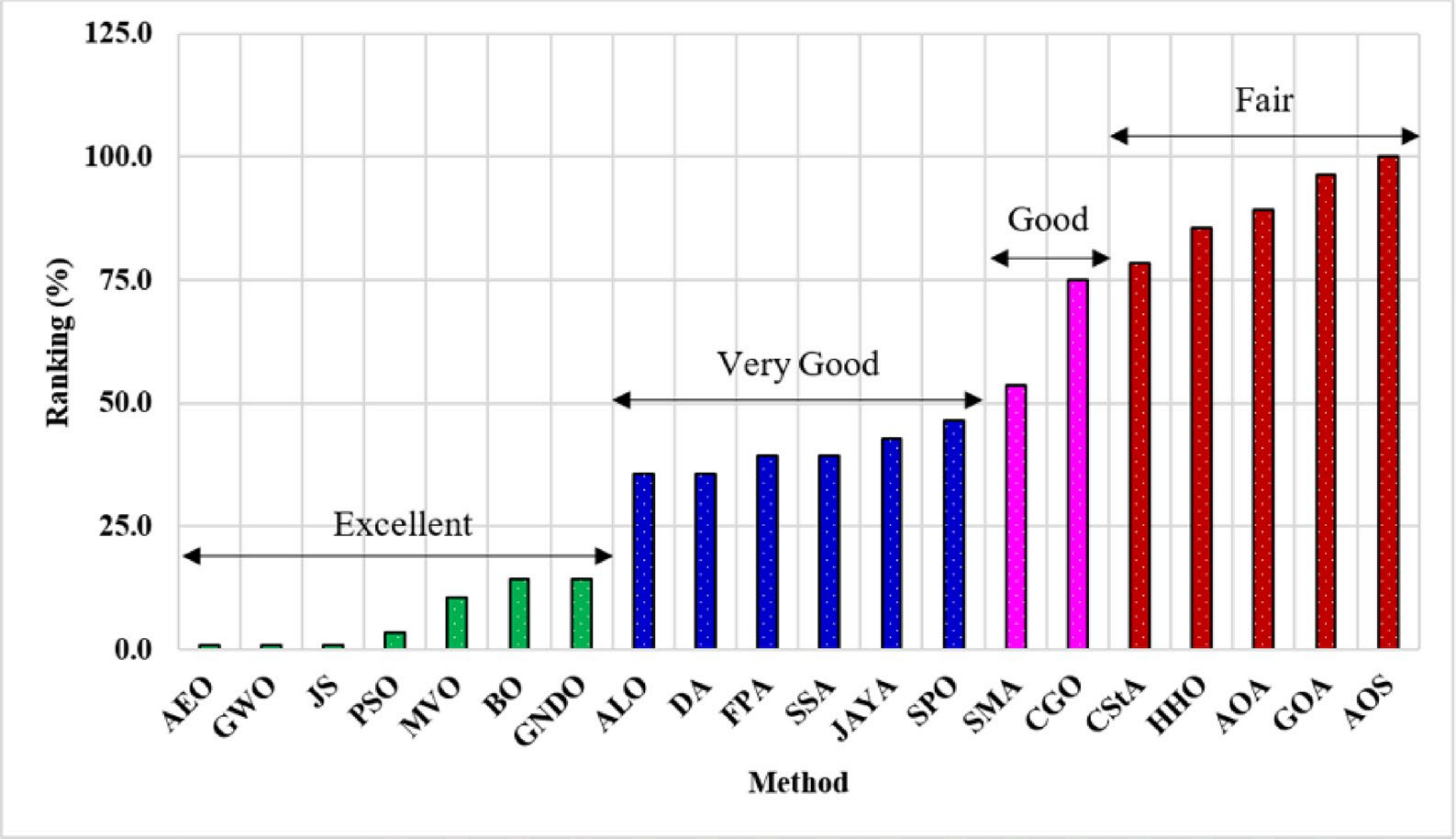
Overall assessment and categories of the optimization methods.

According to the No-Free-Lunch Theorem, all optimization methods perform equally well when their performance is averaged across all potential issues. This was discovered by analyzing the performance of all algorithms. It gives the impression that no one optimization algorithm outperforms all others. If one algorithm performs better than another on a particular category of problems, it will perform worse on a different category of data. The effect of the No-Free-Lunch theorem is applied here in this work; some of the 20 algorithms perform well, and others do not. If A, B, C, and D scores equal 1, 2, 3, and 4, then the scores the algorithm has achieved with all studied systems are summed, as listed in Fig. 26. The total score of each algorithm is obtained as a numerical value, and then its final rank is calculated.

The 20 algorithms are categorized into four classifications depending on their rankings: Excellent, Very Good, Good, and Fair. The 20 algorithms are ordered according to a normalized total achievement score derived from optimizing the 10 studied systems based on the 10 performance parameters. The algorithms AEO, GWO, JS, PSO, MVO, BO, and GNDO rank in the highest or ‘Excellent’ category. The ALO, DA, FPA, SSA, JAYA, and SPO algorithms are classified in the second-best category of ‘Very Good’. SMA and CGO algorithms are ranked third in the ‘Good’ category. The lowest ‘Fair’ algorithms include CStA, HHO, AOA, GOA, and AOS. All algorithms within each category are organized in increasing order (from best to worst).

The current work, which employs 20 optimization algorithms, is compared to published studies for the 33- and 69-bus systems, as shown in Table 12, regarding the power loss values. These two systems are chosen because they are well-known and frequently studied by researchers. For the 33-bus system, the power loss of 87.164 kW in the current work is comparable to or lower than those reported in most published studies. The same trend is observed for the 69-bus system. The power loss in the current work remains fairly stable at 71.644 kW across most optimization methods. These case studies illustrate the behavior of the adopted optimization algorithms within the proposed methodology and demonstrate the effectiveness of the approach in optimizing any distribution system, regardless of size or complexity.

**Table 12:** Comparison of published and current works for the 33- and 69-bus systems.

33-bus system		69-bus system	
Method	Power loss (kW)	Method	Power loss (kW)
Published work			
AEO^59^	87.164	AEO^59^	69.396
HGWO^70^	87.164	MINLP^71^	69.676
PSO^72^	91.300	IA^73^	70.239
MINLP^71^	87.167	HGWO^70^	69.425
EA-OPF^74^	87.17	GAMS^75^	72.090
Hybrid^76^	87.28	MTLBO^77^	69.539
PSO^76^	87.17	HPSO^64^	87.00
IA^73^	87.55	SOS^78^	69.428
BSOA^79^	89.34	BFOA^80^	75.238
Current work			
AEO	87.164	AEO	71.644
GWO	87.164	GWO	71.644
JS	87.164	JS	71.644
PSO	87.164	PSO	71.644
MVO	87.164	MVO	71.644
BO	87.164	BO	71.644
GNDO	87.164	GNDO	71.644
ALO	87.164	ALO	71.644
DA	87.164	DA	71.644
FPA	87.164	FPA	71.741
SSA	87.164	SSA	71.644
JAYA	87.164	JAYA	71.644
SPO	87.164	SPO	71.644
SMA	87.164	SMA	71.644
CGO	87.164	CGO	71.644
CStA	87.164	CStA	71.659
HHO	87.164	HHO	71.651
AOA	87.631	AOA	73.654
GOA	87.166	GOA	71.936
AOS	87.344	AOS	72.193

## Conclusions

The study involves the assessment and categorization of 20 established and innovative optimization algorithms utilized in the optimization of renewable energy sources inside distribution systems. The proposed evaluation method employs the Friedman ranking algorithm across 10 performance metrics, including indices related to power loss, voltage profile, execution time, and frequency of load flow calls. To ensure equitable assessment, all algorithms simulate 10 distribution systems, obtaining ratings depending on their efficacy. All algorithms are systematically categorized as Excellent, Very Good, Good, and Fair. The Excellent category (First-best) has seven algorithms: AEO, GWO, JS, PSO, MVO, BO, and GNDO. The Very Good category includes the ALO, DA, FPA, SSA, JAYA, and SPO algorithms. Additionally, the Good category includes the SMA and CGO algorithms, whilst the lowest category (Fair) consists of the CStA, HHO, AOA, GOA, and AOS algorithms. The proposed evaluation method helps distribution system operators and decision-makers select a suitable optimization methodology tailored to their specific needs and performance objectives. Specific optimization algorithms exhibit suboptimal performance yet still achieve high rankings, as illustrated by GNDO and SPO. Algorithms classified in the Excellent category have low execution durations. Most algorithms invoke the objective function twice. Therefore, the choice of a particular optimization method should not depend solely on one or two objectives.

Future work can include the following recommendations:

- Parameter sensitivity study: This can help in understanding the robustness of the algorithms.
- Real-world application: Validate the performance of the top-ranked algorithms in real-world scenarios to assess their practical applicability and performance under more complex conditions.
- Hybrid algorithms: Explore the potential of hybridizing the top-performing algorithms to leverage the strengths of multiple approaches and potentially achieve enhanced performance.
- Dynamic optimization: Investigate the adaptability of the optimization algorithms to dynamic and changing conditions within distribution systems.

## Data Availability

The data used are included in the article.

## List of symbols

AEO: Artificial ecosystem-based optimization
ALO: Ant lion optimizer
AOA: Arithmetic optimization algorithm
AOS: Atomic orbital search
BESS: Battery energy storage system
BO: Bonobo optimizer
CGO: Chaos game optimization
CStA: Crystal structure algorithm (CryStAl)
DA: Dragonfly algorithm
DG: Distributed generation
DSTATCOM: Distribution static compensator
EVCS: Electric vehicle charging station
FPA: Flower pollination algorithm
GNDO: Generalized normal distribution optimization
GOA: Grasshopper optimization algorithm
GWO: Grey wolf optimizer
HHO: Harris Hawks optimization
JAYA: Enhanced JAYA
JS: Jellyfish search
MO: Multi-objective
MVO: Multi-verse optimizer
PSO: Particle swarm optimization
RES: Renewable energy source
SMA: Slime mould algorithm
SPO: Stochastic paint optimizer
SSA: Salp swarm algorithm
Std: Standard deviation
TVD: Total voltage deviation
TVD_a_: Average total voltage deviation
WT: Wind turbine
f_obj_: Objective function
I_i_: ith branch current
N_b_: Number of system branches
N_C_: Current load flow calling
N_it_: An iteration number
N_itmax_: Maximum number of iterations
N_LF_: Maximum number of load flow calls
N_r_: A run number
N_rmax_: Maximum number of runs
N_rx_: Maximum number of runs (50)
N_s_: Number of system buses
P_i_: ith branch real power
P_L_: Power loss of the total branches
P_L, ave_: Average power loss of all runs
P_L, max_: Maximum power loss of all runs
P_L, min_: Minimum power loss of all runs
Q_i_: ith branch reative power
Q_L_: Reactive loss of the total branches
Q_La_: Average reactive loss
R_i_: ith branch resistance
SI: Stability index
S_i_: ith branch apparent power
SI_a_: Average stability index
V_i_: ith bus voltage
V_L_: Lower (min) voltage
V_La_: Average minimum voltage
V_k_: Voltage at the sending bus k
V_U_: Upper (maximum) voltage
X_i_: ith branch reactance
Z_i_: ith branch impedance
